# Volumetric Engineered 3D Drug Reservoir Against Diabetic Implant Infection via Cuproptosis‐Like Bacterial Death and Hunger‐Triggered Maintenance of Mitochondrial Integrity

**DOI:** 10.1002/advs.202506554

**Published:** 2025-07-03

**Authors:** Dize Li, Hongrui Qin, Min Jiang, Hongjiang Wei, Hongyong Zhao, Ping Tang, Guangyu Jian, Henny C van der Mei, Tao Chen

**Affiliations:** ^1^ Stomatological Hospital of Chongqing Medical University Chongqing Key Laboratory of Oral Diseases and Biomedical Sciences Chongqing Municipal Key Laboratory of Oral Biomedical Engineering of Higher Education Chongqing Medical University Chongqing 401147 P. R. China; ^2^ The Third Hospital of Mianyang Sichuan Mental Health Center Mianyang 621000 P. R. China; ^3^ Department of Biomaterials & Biomedical Technology University of Groningen and University Medical Center Groningen Hanzeplein 1 Groningen 9613 GZ The Netherlands

**Keywords:** carbon quantum dots, cuproptosis, dimensional rise, implant‐associated infection, mitochondria

## Abstract

Implant‐associated infections in diabetic patients pose critical challenges due to immune‐metabolic dysregulation that exacerbates biofilm persistence and tissue damage. This study introduces a “dimensional rise” strategy integrating 3D‐printed porous titanium frameworks with micro‐nano hierarchical structures to establish a mechanically robust, high‐capacity drug reservoir, surpassing the limitations of conventional 2D surface modifications. Copper‐doped carbon quantum dots, synthesized from luteolin, synergize with polydopamine‐mediated photothermal activation to disrupt bacterial copper homeostasis, inducing tricarboxylic acid cycle collapse and cuproptosis‐like death via reactive oxygen species bursts and lipoylated protein aggregation. Concurrently, glucose oxidase depletes local glucose to activate adenosine 5'‐monophosphate‐activated protein kinase phosphorylation in host cells, restoring mitochondrial integrity and metabolic homeostasis through deacetylation. This dual‐action system achieves differential regulation—targeting bacteria while protecting host tissues—and ensures therapeutic coverage across acute infection and chronic healing phases. Validated in three animal models, including Beagles with clinical‐grade implants, the strategy demonstrates potent anti‐biofilm efficacy, prevention of secondary infections, and accelerated diabetic osseogenesis. By upgrading surface engineering to 3D volumetric drug reservoirs, this work establishes a paradigm for differentiated multimodal therapy against implant‐related infections in metabolically compromised hosts, addressing both immediate bactericidal demands and long‐term tissue recovery.

## Graphical abstract

This study provides a volumetric engineered 3D drug reservoir to combat diabetic implant infection. Cu‐doped quantum dots induce cuproptosis‐like bacterial death via metabolic collapse, while glucose oxidase triggers hunger‐triggered mitochondrial protection in host cells. Validated in multi‐model studies, this dual‐mechanism strategy overcomes 2D surface limitations through hierarchical engineering, enabling differential pathogen targeting and tissue preservation.

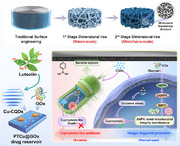

## 1 Introduction

Implant‐associated infections are initiated by biofilm‐forming microbes and lead to persistent inflammation and tissue damage around the implant, thereby affecting the success rate of implantation therapies.^[^ ^1^ ^]^ Under metabolic disease conditions such as diabetes, the risk of infection is heightened due to immune dysregulation and metabolic disorders, resulting in a highly inflammatory microenvironment that further complicates the healing process.^[^ ^2^ ^]^ The underlying causes can be attributed to two triggers of tissue damage: pro‐inflammatory molecules derived from bacteria and uncontrolled inflammatory responses of host immune cells in the context of diabetes, both of which synergistically mediate the chronic pathological state at the inflammation site.^[^ ^3^ ^]^ Therefore, it is vital to treat bacterial infections while maintaining immune homeostasis.

Cuproptosis is a recently identified cell death mechanism.^[^ ^4^ ^]^ It is fundamentally driven by copper (Cu) overload, which leads to cellular function disorder and metabolic suppression.^[^ ^5^ ^]^ Previous studies have revealed that bacteria exhibit a “cuproptosis‐like death” mechanism.^[^ ^6^ ^]^ Under Cu overload, the valence transition between Cu(I) and Cu(II) induces an ROS burst. Furthermore, the tricarboxylic acid (TCA) cycle‐related dihydrolipoamide S‐acetyltransferase (DLAT) binds to copper ions and undergoes abnormal oligomerization, impairing pyruvate synthesis and inhibiting lipoylation. This disrupts the connection between glycolysis and the TCA cycle, ultimately altering metabolic patterns.^[^ ^6^, ^7^ ^]^ Such “cuproptosis‐like” mechanisms integrate ROS bursts with Cu overload‐induced metabolic interference, effectively disrupting the respiratory electron transport chain (ETC) in pathogenic microorganisms and enhancing bactericidal efficacy. However, bacterial Cu resistance, mediated by P1B‐type ATPases, limits their efficacy. P1B‐type ATPases are localized to the cytoplasmic membrane and mediate transmembrane copper efflux, which remains closely tied to bacterial metabolic activity.^[^ ^8^, ^9^ ^]^ Strategies that combine accelerated copper internalization with metabolic interference have emerged as promising multimodal antimicrobial approaches to circumvent copper tolerance and improve ion delivery efficiency. A recent study demonstrated that photothermal effects can disrupt the hydrophobic fragments of phospholipid molecules, whereas thermal enhancement increases membrane fluidity and defect formation, thereby synergistically promoting antibacterial agent internalization.^[^ ^10^ ^]^ Nevertheless, such multimodal strategies face two critical challenges: 1) excessive photothermal effects and Cu delivery may severely damage adjacent normal tissues and cells, and 2) therapeutic efficacy rapidly reduces upon withdrawal of exogenous treatment, allowing surviving bacteria to proliferate and form secondary biofilms. Therefore, to address these issues, two types of “coverage” should be implemented throughout antimicrobial processes: 1) the coverage of different targets, emphasizing differentiated therapeutic strategies to kill pathogens while protecting host cells surrounding the infected areas, and 2) the coverage of different healing stages, requiring sustained delivery of bioactive factors that maintain therapeutic coverage across both early inflammatory and late healing phases after discontinuation of exogenous treatments.

Since the toxic effects caused by Cu accumulation are closely linked to mitochondrial respiration,^[^ ^11^ ^]^ we assumed that maintaining mitochondrial integrity is an effective cell protection strategy. Notably, adenosine monophosphate ‐activated protein kinase (AMPK), the guardian of metabolism and mitochondrial homeostasis, exhibits obvious energy sensitivity and cellular protection capability.^[^ ^12^ ^]^ It can be activated by adenosine triphosphate (ATP) depletion through cellular starvation responses, and the AMPK/SIRT1 pathway can enhance mitochondrial antioxidant capacity by boosting superoxide dismutase and catalase activities.^[^ ^13^, ^14^, ^15^ ^]^ Additionally, AMPK regulates enzymes in the TCA cycle via deacetylation, mitigating copper‐induced lipoylated protein aggregation and maintaining metabolic homeostasis.^[^ ^12^, ^16^ ^]^ Given the prominent pathological features of dysregulated glucose metabolism in diabetes, glucose oxidase (GOx) emerged as an ideal starvation‐inducing trigger that activates the AMPK pathway via glucose consumption.^[^ ^17^, ^18^ ^]^ Therefore, incorporating GOx delivery alongside Cu delivery strategies enables the differential dual regulation of bacteria and host cells, thereby achieving coverage of different intervention targets through metabolic reprogramming and restoration of redox homeostasis.

The potential formation of secondary biofilms by unkilled bacteria poses a long‐term risk of infection during wound healing.^[^ ^19^ ^]^ Therefore, sustained bioactive drug delivery after withdrawing exogenous photothermal stimulation is crucial for prolonged therapeutic effects that can cover both the initial and later healing stages.^[^ ^20^ ^]^ However, surface modifications of implants can only reprogram their properties and load drugs within a limited thickness, resulting in insufficient drug payloads for sustained long‐term therapy.^[^ ^21^, ^22^ ^]^ Additionally, to maintain the initial stability, friction between the implant surface and host tissues during implantation is inevitable, which causes severe coating abrasion and significant loss of loaded drugs.^[^ ^23^ ^]^ To overcome these challenges and achieve the coverage of different healing stages, we propose a novel “dimensional rise” strategy of surface modification, aiming to transform conventional solid titanium implants into mechanically stable porous frameworks, breaking through the thin‐layer limitations of traditional 2D surface treatments. The 3D spatial structure provides augmented drug‐loading interfaces, thereby upgrading surface engineering to volumetric engineering and substantially increasing the drug‐carrying capacity.^[^ ^24^, ^25^, ^26^ ^]^ Furthermore, the 3D trabecular structure can shelter intra‐implant surface coatings from direct contact with host tissues, effectively preventing friction‐induced depletion.^[^ ^27^, ^28^ ^]^ Building on this, our previous study demonstrated that alkali‐heating micropatterning of a titanium (Ti) surface could create micro‐nano hierarchical structures, achieving “secondary dimensional rise” at microscopic levels.^[^ ^29^ ^]^ This dual‐dimensional increase could synergistically expand the drug loading volume for prolonged therapy. Compared to conventional surface modification, the conformation‐enhanced paradigm enables significantly increased drug loading and prolonged controlled drug delivery. Therefore, it meets the critical requirements for long‐term therapeutic efficacy in the clinic.

**Scheme 1:**
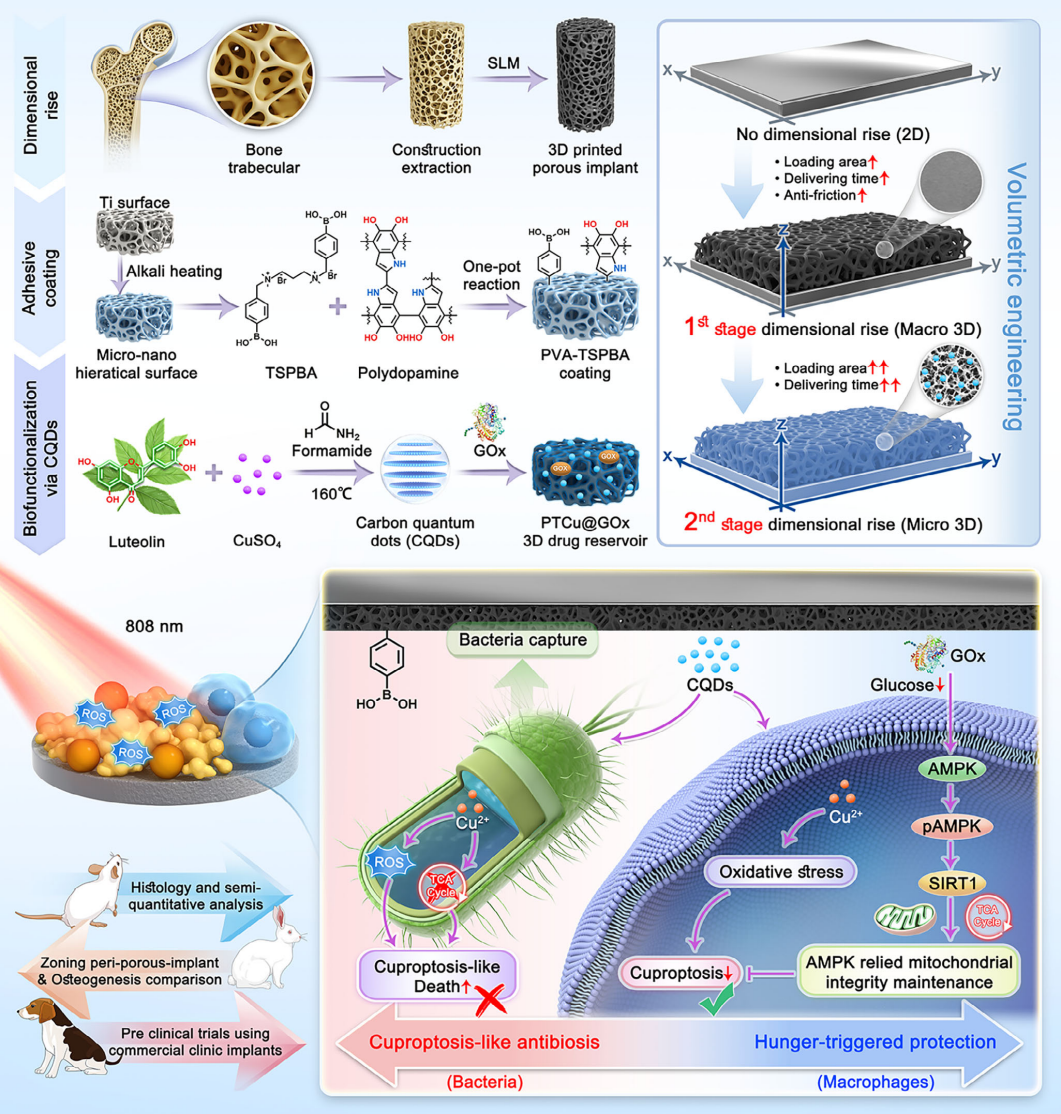
3D drug reservoir creation through dimensional rise, promoting cuproptosis‐like antibiosis and cellular mitochondrial integrity maintenance for self‐protection, achieving ideal management of inflammation against implant‐associated infections.

In this paper, we propose a dimensional rise strategy for titanium implants based on volumetric engineering principles. Using 3D printing technology, we constructed porous architectures to mimic the trabecular bone structure while establishing micro/nano hierarchical structures via alkaline heat treatment, thereby significantly improving the drug‐loading capacity to create a 3D drug reservoir. By leveraging the size‐dependent biofilm penetration and catalytic potential of carbon quantum dots,^[^ ^30^ ^]^ we synthesized copper‐doped carbon quantum dots (Cu‐CQDs) from luteolin. A multifunctional adhesive coating incorporating polydopamine (PDA) and N^1^‐(4‐boronobenzyl)‐N^3^‐(4‐boronophenyl)‐N^1^,N^1^,N^3^,N^3^‐tetramethylpropane‐1,3‐diaminium (TSPBA) was designed to simultaneously load Cu‐CQDs and GOx to enable differential therapeutic effects on bacterial and host cells. The photothermal properties of PDA enhance the phospholipid bilayer fluidity, facilitating copper ion penetration and subsequent bacterial copper overload.^[^ ^10^, ^31^ ^]^ Notably, we observed disruption of the TCA cycle and cuproptosis‐like effects in copper‐overloaded bacteria. Concurrently, GOx maintained mitochondrial integrity through an AMPK‐dependent mechanism, thereby providing cytoprotective effects. We also built three animal models and confirmed sustained therapeutic efficacy against implant‐associated infections by applying this strategy to commercial clinical implants (**Scheme** **1**). In summary, by integrating 3D‐printed macroporous frameworks with alkali‐heated micro/nanoarchitectures, we constructed a hierarchical drug reservoir. This spatial design was further functionalized with Cu‐CQDs and GOx, enabling spatiotemporal control of copper toxicity and metabolic reprogramming. This strategy upgrades the traditional modified surface into a 3D drug reservoir, thereby satisfying both anti‐abrasion requirements and long‐term drug delivery needs and achieving coverage at all healing stages. It enables differentiated control of copper toxicity and metabolic reprogramming, coupling localized antibacterial action with sustained mitochondrial protection. Consequently, our study represents a promising paradigm shift in the multimodal treatment of implant‐related infections in patients with diabetes.

## 2 Results and Discussion

### 2.1 Volumetric Engineering with the Dual Dimensional Rise Implant Strategy and Fabrication of the PTCu@GOx Drug Reservoir

**Figure 1:**
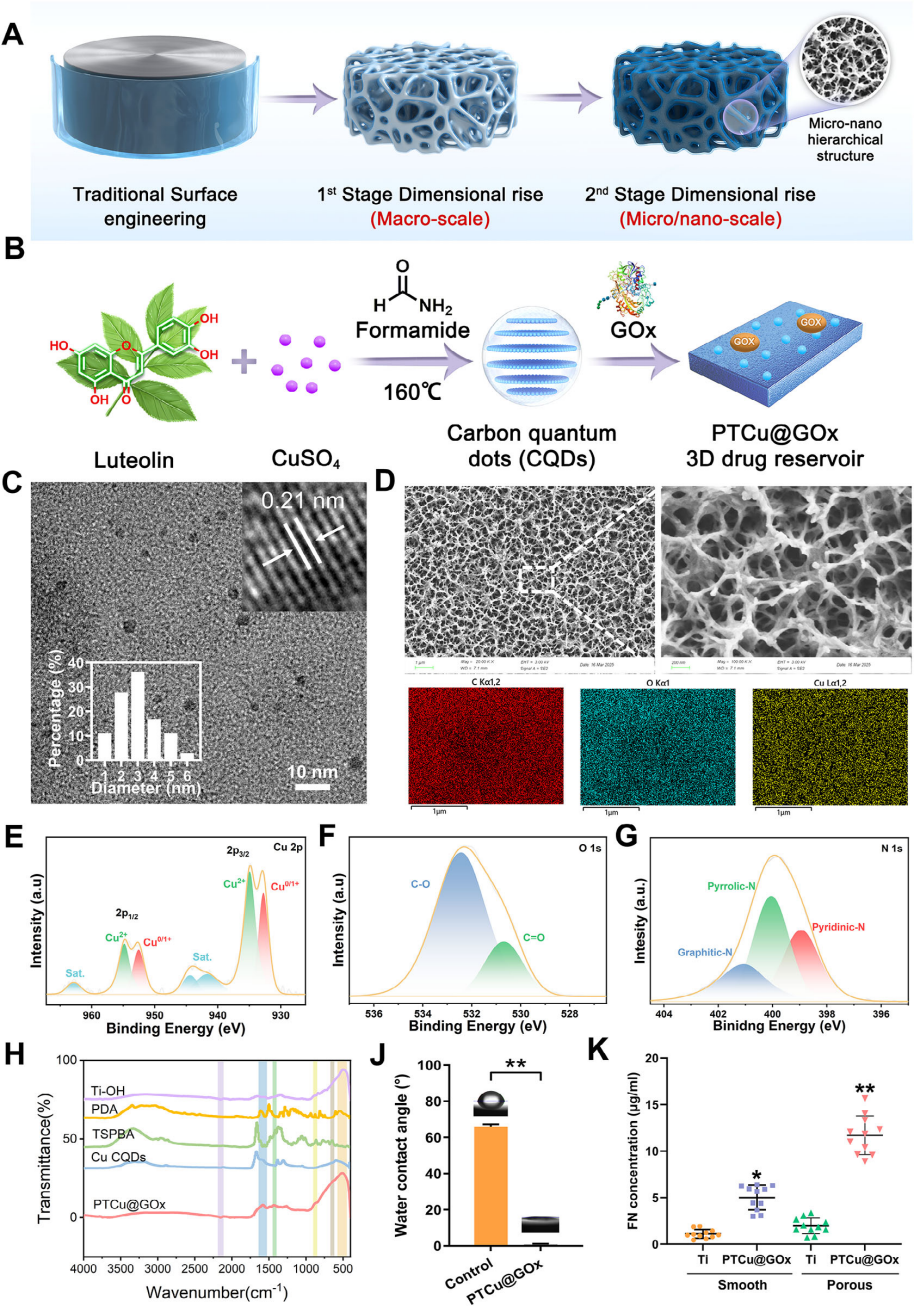
Dual‐dimensional rise of the implant surface and fabrication of PTCu@GOx. A) Schematic illustration of dual dimensional rise from surface engineering to volumetric engineering; B) Synthesis of CQDs; C) TEM observation (Scale bar = 50 nm); D. SEM and EDS analysis of the implant surface with PTCu@GOx; E–G) High‐resolution XPS analysis of the implant surface after volumetric engineering; H) FTIR analysis confirming successful fabrication of PTCu@GOx coating; J) Contact angle measurement of coating (*n* = 3); K) Protein adsorption assay on smooth and porous Ti discs (*n* = 3, *p < 0.05; **p < 0.01, one‐way ANOVA with SNK post hoc test).

Trabecular bone‐mimetic porous titanium implants were fabricated based on previously reported additive manufacturing techniques.^[^ ^29^ ^]^ Briefly, a cylindrical structure (ϕ = 3 mm × 10 mm) was digitally designed with biomimetic trabecular porosity using computer‐aided design and printed via selective laser melting using grade‐4 commercially pure titanium powder. The implants had an average pore size of 385.02 ± 1.38 µm, trabecular strut diameter of 200.05 ± 0.67 µm, and porosity of 76.82% ± 0.83%. Compressive strength analysis showed that the porous Ti implants exhibited an elastic modulus comparable to that of the cancellous bone, demonstrating sufficient mechanical load‐bearing capacity. Additionally, high‐resolution micro‐computed tomography (CT) measurements and calculations revealed that the dimensional rise strategy increased the volumetric specific surface area (SSA_v_) from 1.58 mm^−1^ (solid Ti) to 5.65 mm^−1^ (porous Ti, Figure S1, Supporting Information), achieving an enhancement factor of ≈3.57‐fold. This dimensional rise significantly increased the surface‐accessible area available for functional modification. Subsequently, alkali‐heat treatment was employed to create surface micro‐nano hierarchical structures, enabling “2nd stage dimensional rise” at the microscale. This hierarchical topography further increased the SSA_v_ by over fivefold, substantially improving the drug‐loading capacity and sustained‐release kinetics of the coating‐based tissue engineering strategies (**Figure** **1**A). The essence of this dimensional expansion strategy lies in the evolution from “interface modification” to “volumetric design”—a 3D reconstruction spanning macro‐ and microstructural scales through volumetric engineering.^[^ ^32^ ^]^ This approach overcomes the inherent limitations of conventional surface engineering (i.e., thin‐layer constraints) and demonstrates revolutionary potential for bone‐repair applications that require long‐term drug delivery and multifunctional bioactivity.

Cu‐CQDs derived from luteolin were synthesized using a one‐pot hydrothermal method (Figure 1B).^[^ ^33^ ^]^ Morphological characterization was performed using transmission electron microscopy (TEM), and the Cu‐CQDs exhibited a uniform spherical morphology with an average diameter of 3.44 ± 1.19 nm (Figure 1C). Lattice fringes of 0.21 nm may arise from the formation of Cu─C bonds introduced by Cu doping as well as local compressive stress in the carbon layers resulting from the coordination between Cu and oxygen‐containing functional groups on the surface of the CQDs. X‐ray photoelectron spectroscopy (XPS) analysis of the CQDs showed that the proportion of Cu^+^ to Cu^2+^ was 2.03:1, indicating the potential of the catalytic properties based on valence state changes (Figure S2, Supporting Information). Upon immobilization onto the alkali‐heat‐treated Ti‐OH substrates, the homogeneous distribution of Cu across the micro/nano hierarchical structures was confirmed by elemental mapping, indicating the successful loading of the Cu‐CQDs (Figure 1D). The XPS results further demonstrated that the characteristic peaks of the Cu‐CQDs were retained after loading onto the Ti sheets, further indicating their successful loading and conferred catalytic potential (Figure 1E). Additionally, characteristic peaks corresponding to the C═O bonds (287.8 eV) of luteolin and N doping were observed. Notably, the dominant pyrrolic N configuration (400.1 eV, 68% of total N content) indicated the formation of a five‐membered aromatic ring structure, where N atoms bonded to two carbon atoms via σ‐bonds and participated in π‐conjugation—a feature consistent with PDA‐like architectures (Figure 1F,G; Figure S3, Supporting Information).^[^ ^34^ ^]^ X‐ray diffraction analysis confirmed that the incorporation of Cu‐CQDs did not significantly alter the crystalline phase of the titanium substrate, while Fourier Transform Infrared Spectroscopy identified characteristic absorption bands of Cu‐CQDs (C═O stretching at 1640 cm⁻¹) and PDA (N‐H bending at 1540 cm⁻¹), confirming the successful fabrication of the PTCu@GOx composite coating (Figure 1H; Figure S4, Supporting Information). Remarkably, the PTCu@GOx coating reduced the water contact angle from 85.3° (bare porous Ti) to 12.5°, demonstrating a significantly enhanced hydrophilicity (Figure 1J). To evaluate the biological implications of the dual‐dimensional rise strategy, protein adsorption assays were conducted on smooth Ti discs and 3D‐printed porous Ti substrates with and without coatings. The results revealed that 1st stage dimensional rise (3D porous architecture) moderately increased the protein adsorption capacity, whereas the synergistic integration of 2nd stage dimensional rise (micro/nano hierarchical surfaces) further amplified this capability by six‐fold (Figure 1K).^[^ ^35^ ^]^ This outcome underscores the profound impact of hierarchical volumetric engineering on drug loading and subsequent modulation of biological interactions.

### 2.2 PTCu@GOx Coating Exhibits Multiple‐Type ROS Generation, Stable Photothermal/Photodynamic Therapy, and Enhanced Tribological Resistance

**Figure 2:**
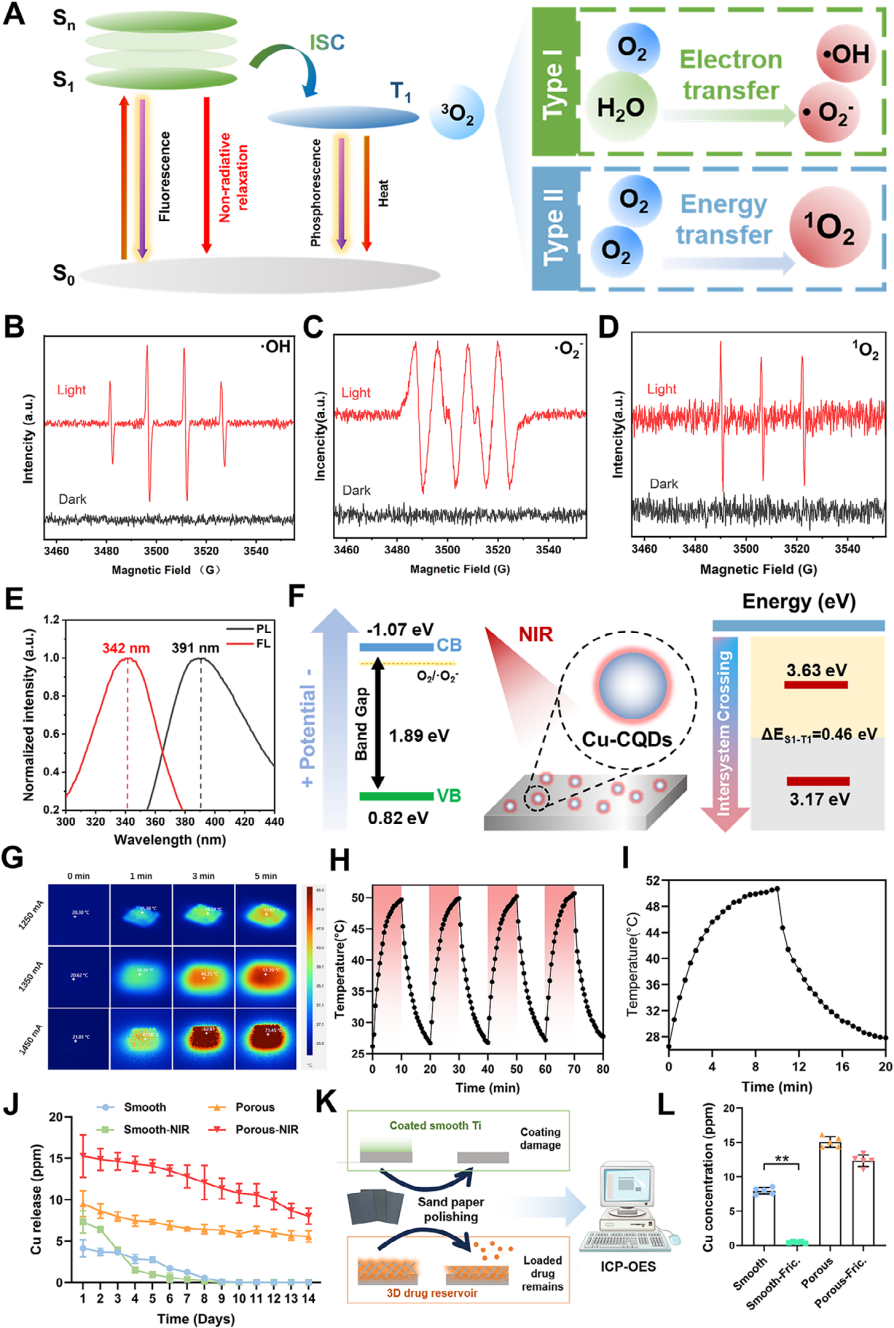
Photocatalytic, photothermal, and anti‐friction properties of the PTCu@GOx drug reservoir. A–C) ESR detection of ROS generation; D) fluorescence and phosphorescence detection; E) ISC mechanism and ROS generation pathways; H–J) PTT effect evaluation and switch on/off assay (*n* = 6); K) Drug release profiles of smooth and porous Ti disks with PTCu@GOx under PTT stimulation; L,M) Anti‐friction performance and drug retention ability of dimensional rise PTCu@GOx drug reservoir (*n* = 3, *p < 0.05; **p < 0.01, one‐way ANOVA with SNK post hoc test).

The presence of mixed‐valence Cu species in Cu‐CQDs endows the material with catalytic potential, which is further enhanced under near‐infrared (NIR) irradiation.^[^ ^36^ ^]^ Under NIR activation, electrons can be excited to the singlet excited state (S₁), followed by non‐radiative relaxation to generate heat, thereby inducing a photothermal effect.^[^ ^37^ ^]^ Additionally, electrons undergoing intersystem crossing populate the triplet state (T₁) and subsequently produce ROS via two distinct mechanisms: i) Type I mechanism—electron transfer between T₁ and surrounding H₂O and O₂ to produce •O₂⁻ and •OH; ii) Type II mechanism—energy transfer from T₁ to ground‐state oxygen (^3^O₂) to generate ¹O₂ (**Figure** **2**A).^[^ ^38^ ^]^ To further confirm this, electron spin resonance (ESR) analysis was carried out and revealed that NIR triggered the generation of multiple ROS, including •OH, •O₂⁻, and ¹O₂, on the PTCu@GOx‐coated porous titanium surface (Figure 2B–D). To elucidate the mechanism of ROS generation, fluorescence and phosphorescence emission spectra were analyzed. Fluorescence and phosphorescence emission peaks were observed at 342 and 391 nm, respectively. Using the relationship E = 1240/λ (eV), the energy levels of S₁ and T₁ were calculated as 3.63 and 3.17 eV, respectively, yielding a singlet‐triplet energy gap (ΔES₁‐T₁) of 0.46 eV (Figure 2E,F). Furthermore, XPS valence band analysis indicated that the valence band of PTCu@GOx was positioned at 0.82 eV. The maximum position of this valence band indicates a pronounced hole‐oxidation capability of PTCu@GOx. Additionally, the ultraviolet‐visible absorption spectra demonstrated strong NIR absorption in the 800–1000 nm range, with a peak at 808 nm. The optical bandgap, calculated using the Tauc plot, was determined to be 1.89 eV (Figure S5, Supporting Information).^[^ ^39^ ^]^ Therefore, the minimum conduction band was calculated to be −1.07 eV. Additionally, the narrow bandgap facilitates efficient photoinduced electron transitions under NIR excitation, which is consistent with the observed catalytic activity (Figure 2F).

Owing to the vibrational relaxation capabilities of polydopamine and CQDs, PTCu@GOx exhibits pronounced photothermal performance under NIR activation, where non‐radiative relaxation dominates after electron excitation to the excited state (Figure S5, Supporting Information).^[^ ^40^, ^41^ ^]^ As shown by photothermal temperature detection with 1350 mA NIR stimulation, the temperature could reach 51.29 °C within 5 min, accompanied by a stable and pronounced “on/off switching effect” (Figure 2G–I), demonstrating precise controllability of the photothermal response. The photothermal conversion efficiency (η) was c44.2%, primarily attributed to the broad‐spectrum absorption (400–1100 nm)^[^ ^42^ ^]^ and high molar extinction coefficient (8.2 × 10⁸ cm⁻¹·M⁻¹ at 808 nm)^[^ ^43^ ^]^ of PDA (Figure S6, Supporting Information). As mentioned before, the conjugated π‐electron system of PDA facilitated the rapid relaxation of photoinduced electron‐hole pairs through non‐radiative transitions, thereby efficiently converting photon energy into heat.^[^ ^43^ ^]^ The 3D structure of porous Ti also increased the loading area of Cu ions, thereby significantly increasing the Cu delivery ability of PTCu@GOx under NIR irradiation (Figure 2J). This volumetric engineering‐based 3D structuring overcomes the limitations of thin‐film configurations and significantly prolongs the duration of photothermally triggered drug release, thereby satisfying the demands of broader biomedical applications.

Notably, although existing studies rarely address the tribological resistance of bioactive coatings, clinical implantation inevitably subjects the implant surface to friction against the host bone tissue, which may mechanically compromise drug‐loaded coatings and undermine their therapeutic efficacy.^[^ ^28^ ^]^ To overcome this limitation, trabecular bone‐mimetic 3D‐printed porous titanium implants have been designed to decouple the coating integrity from implantation‐induced friction. Specifically, the hierarchical architecture confines the PTCu@GOx coating primarily within the internal pore walls (non‐contact regions during insertion), thereby preserving the “drug reservoir” functionality. To validate this hypothesis, in vitro abrasion tests were performed by applying 500 N cyclic friction (simulating surgical insertion forces) using 400‐grit sandpaper on smooth and porous Ti disks coated with PTCu@GOx. Using ICP‐OES, we observed that the loaded drug volume on smooth Ti disks was significantly decreased after friction (*p* < 0.01). In comparison, in porous Ti disks, no significant difference of the drug volume was observed before and after friction (*p* > 0.05), meaning that the porous structure could protect loaded drugs from exogenous friction (Figure 2K,L). These results demonstrate a critical advantage of the dimensional rise strategy: structural confinement of functional coatings minimizes mechanical degradation during implantation, ensuring long‐term localized therapeutic delivery to combat infection risks and support tissue regeneration.

### 2.3 PTCu@GOx Enhances Bacterial Cu Overload Through Photothermally Stimulated Cell Membrane Damage

**Figure 3:**
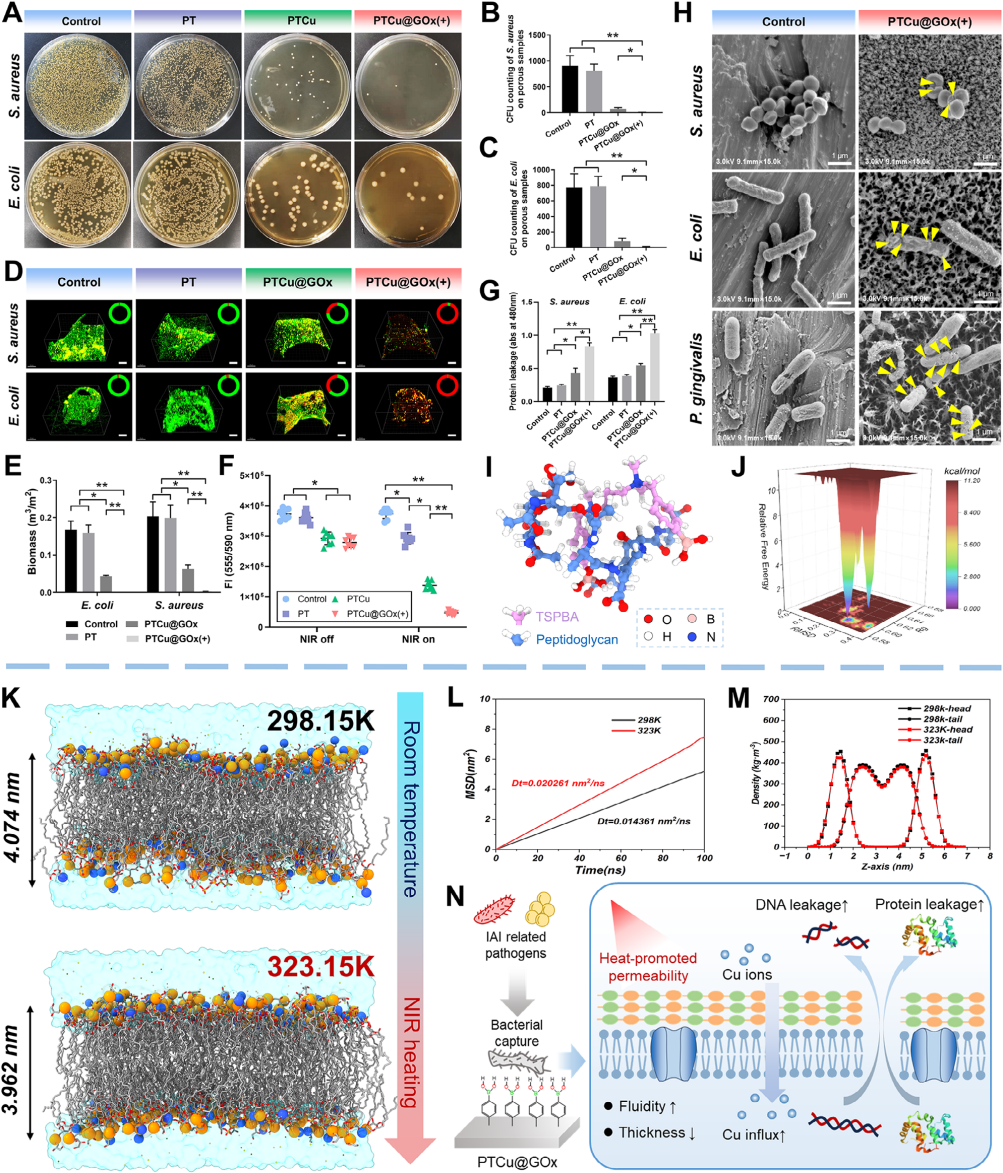
Antibacterial properties of PTCu@GOx and photothermal‐enhanced copper internalization mechanisms. *S. aureus*, *E. coli*, and *P. gingivalis* were cultured with PTCu@GOx for further study. A–C) Leachable antibacterial activity via agar plating and colony‐forming unit (CFU) counts showing its leachable antibacterial capacity (*n* = 3); D,E) Biofilm live/dead staining and semi‐quantification showing its contact antibacterial capacity (Scale bar = 50 µm, *n* = 3); F) Alamar Blue assay exhibiting bacterial metabolic inhibition (*n* = 6); G,H) Protein leakage and SEM imaging to observe the damage to bacterial membranes (Scale bar = 1 µm); I,J) Molecular docking of N^1^‐(4‐boronobenzyl)‐N^3^‐(4‐boronophenyl)‐N^1^,N^1^,N^3^,N^3^‐tetramethylpropane‐1,3‐diaminium (TSPBA) with peptidoglycan showing the bacterial capture capacity; K) Molecular dynamics simulations of lipid bilayers showing the POPG bilayer snapshots at 298 and 323 K; L) MSD comparison; M) Lipid density distribution. N) Schematic of photothermal‐enhanced membrane permeability. (*p < 0.05; **p < 0.01, one‐way ANOVA with SNK post hoc test).

PTCu@GOx, benefiting from its bacterial capture properties, effects of photothermal therapy (PTT), and the chemodynamic features of CQDs, has potent broad‐spectrum antibacterial potential against various bacterial strains. To evaluate the antibacterial capability of PTCu@GOx, its leachability and contact antibacterial properties were assessed. For leachable antibiosis, counting of colony‐forming units (CFUs) showed a significant reduction in CFUs for both *S. aureus* and *E. coli* (*p* < 0.01), which was further confirmed using the inhibition zone assay (**Figure** **3**A–C; Figures S7,S8, Supporting Information). For contact antibiosis, bacteria were directly inoculated onto the coated porous titanium discs for co‐cultivation. Live/dead staining revealed negligible antibacterial activity for the pure PT coating, whereas PTCu@GOx with Cu‐CQDs partially killed bacteria without NIR stimulation, which was further strengthened by NIR stimulation (Figure 3D). Additionally, the biomass of the *S. aureus* biofilm was reduced from 0.203 ± 0.039 to 0.0034 ± 0.0003 (*p *< 0.01), and the *E. coli* biofilm biomass decreased from 0.168 ± 0.023 to 0.0011 ± 0.0001 (*p *< 0.01), indicating the remarkable anti‐biofilm properties of PTCu@GOx under NIR. Petrifilm analysis demonstrated that PTCu@GOx exhibited 45.16% ± 3.67% contact antibacterial activity against *S. aureus* on the agar film in contact with the coated titanium disc, which increased to 96.08% ± 3.22% under NIR stimulation; similarly, the coating showed a 47.01% ± 5.65% antibacterial rate against *E. coli*, which was enhanced to 96.68% ± 4.42% with NIR (Figure S9, Supporting Information). These results were further confirmed by Alamar Blue staining, which indicates bacterial metabolic activity, showing that PTCu@GOx(+) reduced the metabolic capacity of bacteria by 89.12% ± 5.23%, highlighting the significant antibacterial ability of the coating (Figure 3F).

We also confirmed bacterial cell membrane disruption by detecting protein and nucleic acid leakage. Results indicated that under the influence of PTCu@GOx, the optical density values for protein leakage in *S. aureus* increased from 0.211 ± 0.016 to 0.832 ± 0.055, while those for *E. coli* increased from 0.366 ± 0.019 to 1.032 ± 0.051. Similarly, nucleic acid leakage analyses revealed that the extracellular DNA concentration in the photothermally triggered PTCu@GOx group rose by 4.47‐fold for *S. aureus* and by 15.06‐fold for *E. coli* (Figure 3G; Figure S10, Supporting Information). These data collectively suggest that photothermally activated PTCu@GOx substantially promotes cell membrane damage, leading to the leakage of intracellular contents. Scanning electron microscopy (SEM) was conducted to further confirm the damage to the cell membrane. The images revealed notable outer membrane defects in *S. aureus* and *E. coli* treated with PTCu@GOx combined with NIR irradiation, with significant bacterial morphological changes such as wrinkling and perforation, indicating compromised cell membrane integrity (Figure 3H). These findings corroborated the results of the DNA and protein leakage experiments. To demonstrate the antibacterial efficacy of the coating in peri‐implantitis, *P. gingivalis*, the dominant bacterium in peri‐implantitis, was also tested for antimicrobial activity and cell membrane damage (Figure 3H; Figure S10, Supporting Information).^[^ ^44^ ^]^ The results showed that under NIR exposure, PTCu@GOx exhibited the same significant bactericidal effect against *P. gingivalis*, indicating its potential application in combating oral implant‐related infections.

The highly efficient bactericidal action of the coating can also be attributed to the inclusion of TSPBA in the fundamental molecular framework, which facilitates bacterial capture via boronate‐ester bond formation. Our molecular docking analysis revealed that during the 100 ns simulation, TSPBA exhibited stable binding activity with peptidoglycan monomers (Figure 3I,J). This can be ascribed to the reactive structure of the four‐armed phenylboronic acid, which comprises a boron center linked to hydroxyl groups capable of forming covalent boronate esters with diol‐containing components.^[^ ^45^ ^]^ Given that bacterial cell walls and the matrix of the biological membrane are rich in polysaccharide components containing diols, TSPBA demonstrates binding activity toward bacterial surfaces. This underpins the ability of PTCu@GOx to capture bacteria and exert efficient bactericidal action.

To provide further evidence for the photothermal effect on the disruption of the phospholipid bilayer of bacterial cell membranes, we employed all‐atom molecular dynamics simulations to analyze changes in the atomic permeability of the bacterial cell membrane under the influence of PTT. The phospholipid bilayer, a crucial scaffold for bacterial structural stability, was modeled using the CHARMM‐GUI tool to obtain the topology of the bilayer at two different temperatures: 25 °C (298.15 K) and 50 °C (323.15 K).^[^ ^46^ ^]^ The lipid composition was set to 60% POPG, 15% POPE, 15% DOTAP, and 10% TOCL, with each leaflet containing 160 lipid molecules for a total of 320 molecules.^[^ ^47^ ^]^ Each side of the membrane was flanked by a 17.5 Å water layer (comprising 32,133 atoms), and the system was balanced with 234 sodium ions and 26 chloride ions for osmotic equilibrium. The simulation results indicated increased lipid mobility at 323.15 K due to the elevated kinetic energy of the lipid molecules at higher environmental temperatures, which significantly enhanced the lateral diffusion potential of the membrane. Analysis of the root‐mean‐square displacement of the phospholipid head atoms (Dt) demonstrated that at 323.15 K, the bilayer exhibited a distribution of points with higher diffusion coefficients on the xy‐plane, with the membrane Dt at high temperature being 0.020261 nm^2^/ns, exceeding the Dt value at 298.15 K (0.014361 nm^2^/ns). This finding suggests that the fluidity of the bilayer substantially increased, whereas its thickness decreased with heating (Figure 3K,L; Figure S11, Supporting Information). Additionally, the lipid order parameter, an indicator of lipid rigidity, showed decreased rigidity in the hydrophobic tails of the phospholipids at 323.15 K, indicating increased flexibility and facilitating the internalization of external ions (Figure 3M).^[^ ^10^ ^]^ In summary, the thermal effect enhanced the fluidity and presence of defects in the cell membrane, synergistically augmenting the internalization of drugs from PTCu@GOx and the antibacterial function (Figure 3N).

### 2.4 PTCu@GOx Induces Cuproptosis‐Like Antibiosis

**Figure 4:**
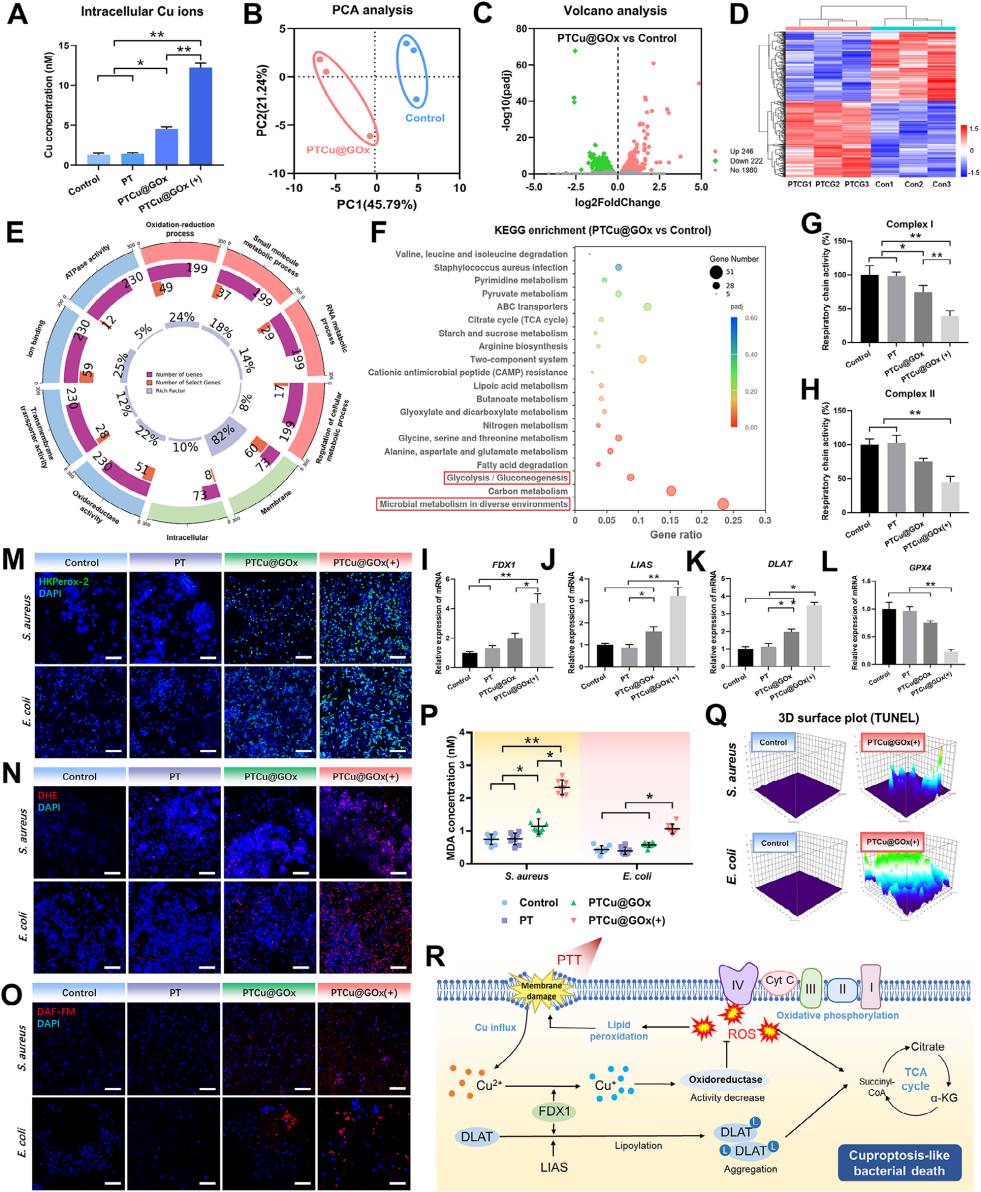
PTCu@GOx‐induced copper overload, leading to cuproptosis‐like death through metabolic dysfunction and lipid peroxidation. A) ICP‐OES analysis of intracellular copper overload in *MRSA* under NIR stimulation; B) Principal component analysis of *MRSA* transcriptome; C,D) Volcano plot and hierarchical clustering of differentially expressed genes showing PTCu@GOx affects the gene expression pattern change; E,F) GO and KEGG enrichment analysis of metabolic pathways; G,H) Enzyme activity assays of ETC complexes I and II (*n* = 3); I–L) qRT‐PCR validation of cuproptosis‐related markers (*GPX4, LIAS, DLAT, FDX1, n* = 3); M–O) ROS/RNS detection with fluorescent probes (Scale bar = 10 µm); P) MDA assay for lipid peroxidation (*n* = 6); Q) TUNEL assay detecting bacterial apoptosis; R) Schematic of cuproptosis‐like death mechanism. (*p < 0.05; **p < 0.01, one‐way ANOVA with SNK post hoc test).

To further validate the internal biochemical reactions and ascertain the copper‐related antimicrobial mechanism, methicillin‐resistant *S. aureus* (*MRSA*) was treated with PTCu@GOx as a representative drug‐resistant bacterial strain. We first observed the intra‐cellular Cu concentration using ICP‐OES, and the results indicated an overload of Cu in PTCu@GOx‐treated *MRSA* (**Figure** **4**A). Prokaryotic transcriptomic analysis was performed. The PCA indicated suitable consistency within group variance, confirming the validity of the RNA‐Seq results (Figure 4B). Volcano plots and cluster analysis revealed that PTCu@GOx + NIR upregulated 1182 genes and downregulated 1266 genes within the *MRSA* biofilm compared to the control group (Figure 4C,D). Furthermore, Kyoto Encyclopedia of Genes and Genomes (KEGG) and gene ontology (GO) enrichment results showed that differentially expressed genes (DEGs) in the PTCu@GOx group were enriched in metabolism related events, suggesting that the photothermally activated PTCu@GOx‐induced antibiosis of *MRSA* may stem from its interference with metabolic activities, including the TCA cycle (Figure 4E,F).

Cellular cuproptosis is reportedly distinct from known death mechanisms and depends on obstructed cellular respiration, disordered TCA cycle,^[^ ^48^ ^]^ impaired Fe‐S cluster synthesis,^[^ ^49^ ^]^ and accumulation of lipid‐acylated proteins.^[^ ^5^ ^]^ Therefore, we hypothesized that copper overload‐induced ‘copper death’ in bacteria shares homology with that in eukaryotic cells. Hence, we assessed the activities of metabolic complexes I and II in *MRSA*, finding that the activity of complex I was reduced to 39.26% ± 7.64%, and complex II activity decreased to 44.56% ± 8.59% (p < 0.01), further illustrating that PTCu@GOx‐mediated copper overload can block bacterial metabolic pathways (Figure 4G,H). Next, cuproptosis marker expression was evaluated using quantitative reverse transcription‐polymerase chain reaction (qRT‐PCR), showing that *FDX1*, *LIAS*, and *DLAT* significantly increased in PTCu@GOx(+) compared to the control group (*p* < 0.05, Figure 4I–L). When copper ions are internalized, the upregulated FDX1 converts Cu^2^⁺ into the more toxic Cu⁺, while increased LIAS expression promotes lipoylation modification of DLAT, thereby inducing its insoluble aggregation and mediating bacterial cell death.^[^ ^50^, ^51^ ^]^ Additionally, decreased levels of GPX4, an antioxidant factor, may result from Cu‐induced interference with the metabolic pathways involved in the synthesis of related factors. Notably, the synthesis of Fe‐S clusters is closely linked to the internal utilization of active copper ions by the bacteria.^[^ ^49^ ^]^ Therefore, we performed qRT‐PCR for *sufB* and *sufC*, the key genes involved in Fe‐S cluster generation,^[^ ^52^, ^53^ ^]^ and observed that PTCu@GOx significantly decreased their expression (Figure S12, Supporting Information). To further validate the cuproptosis‐like death in bacteria, we performed Western blot analysis of DLAT oligomerization in MRSA, which revealed enhanced oligomer formation in the PTCu@GOx‐treated group compared to controls (Figure S13, Supporting Information). Additionally, ATP level assays were conducted to assess the impact of PTCu@GOx on bacterial energy metabolism. Significant ATP decrease (*p* < 0.05) was observed in S. aureus, E. coli, and P. gingivalis following PTCu@GOx treatment (Figure S14, Supporting Information), corroborating the disruption of metabolic homeostasis. Cu overload‐induced oxidative stress is another mechanism underlying the antibacterial function of PTCu@GOx. We comprehensively assessed internal oxidative stress levels in bacteria using different ROS probes. The results demonstrated that even without NIR, PTCu@GOx mediated an increase in the levels of H_2_O_2_, superoxide anions, and reactive nitrogen species, and this trend was significantly intensified by NIR stimulation (Figure 4M–O; Figure S15, Supporting Information). Since this pathological process is highly associated with lipid overoxidation, malondialdehyde (MDA) analysis was performed, and we found that PTCu@GOx mediated a significant increase in MDA within the bacterial biofilm (Figure 4P). Finally, bacterial apoptotic markers, as indicated by the terminal deoxynucleotidyl transferase dUTP nick end labeling (TUNEL) assay, showed an upward trend, consistent with the increases in ROS generation and MDA (Figure 4Q; Figure S15, Supporting Information). These findings suggest that the PTCu@GOx coating could be a potent inducer of bacterial cuproptosis through metabolic disorders. It causes oxidative stress, which strengthens membrane damage and disrupts metabolic processes. Meanwhile, Cu internalization led to DLAT aggregation and activated cuproptosis‐like bacterial death (Figure 4R).

In summary, our omics analysis and assessment of related biomarkers collectively suggest that the PTCu@GOx coating can specifically disrupt the bacterial TCA cycle and amplify lipid peroxidation through different types of ROS. These mechanisms are comparable to those of cuproptosis in eukaryotic cells. Consequently, we propose that the bacterial Cu overload induced by PTCu@GOx results in an efficient bactericidal effect based on a Cu death‐like mechanism.

### 2.5 PTCu@GOx Promotes Hunger‐Triggered Antiinflammation via the pAMPK Pathway

**Figure 5:**
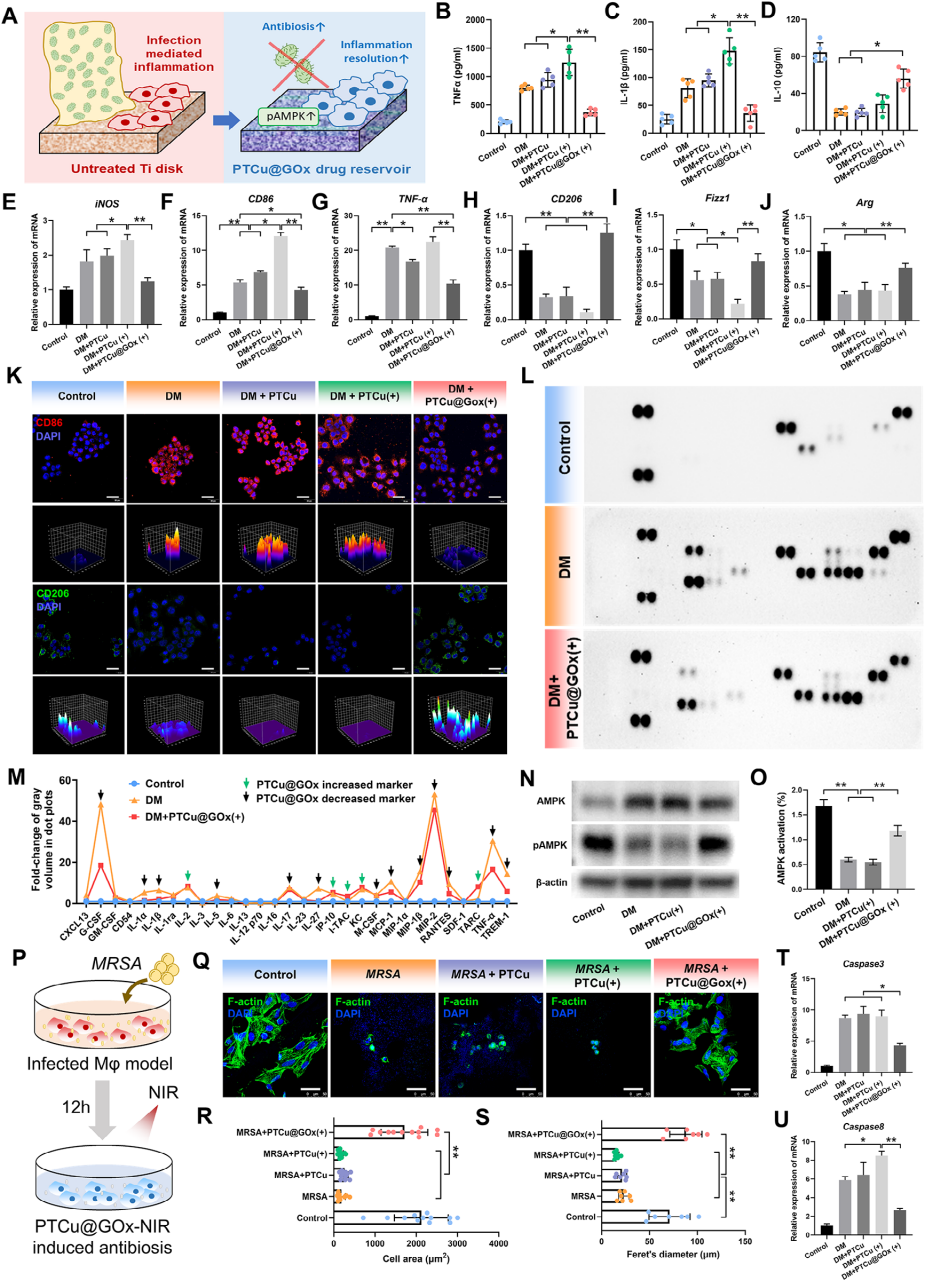
PTCu@GOx‐mediated AMPK phosphorylation counteracts bactericidal side effects and promotes macrophage inflammation resolution. A) Schematic of bactericidal activity with cytoprotective function; B–D) Macrophage inflammatory cytokine profiling by ELISA (*n* = 5); E–J) qRT‐PCR analysis of pro‐inflammatory macrophage markers (*iNOS, CD86, TNF‐α*) and anti‐inflammatory markers (*CD206, Arg1, Fizz1*) (*n* = 3); K) Immunofluorescence staining of CD86 (red) and CD206 (green) (Scale bar = 100 µm); (L‐M) Dot blot analysis of the inflammatory spectrum of macrophages; N,O) Western blot of AMPK/p‐AMPK levels; P) Schematic of direct bacteria‐macrophage co‐culture system; Q–S) Phalloidin staining of macrophage adhesion and spreading (Scale bar = 50 µm); T–U) qRT‐PCR of apoptosis‐related markers (*n* = 3). (*p < 0.05; **p < 0.01, one‐way ANOVA with SNK post hoc test).

Host cells and pathogenic microorganisms dynamically coexist. Macrophages play a pivotal role in the host's defense against infections by recognizing, phagocytosing, and eliminating pathogenic microorganisms.^[^ ^54^ ^]^ These cells can polarize into pro‐ or anti‐inflammatory phenotypes in response to tissue infections, thereby modulating their activity and functionality.^[^ ^55^ ^]^ However, the efficient delivery of Cu^2^⁺ by PTCu@GOx may inadvertently lead to excessive intracellular Cu accumulation within host cells, triggering bactericidal side effects and promoting inflammatory phenotypic transitions driven by bactericidal overload. Such excessive pro‐inflammatory polarization can escalate the secretion of pro‐inflammatory mediators such as IL‐1β and TNF‐α, potentially inducing a cytokine storm (**Figure** **5**A).^[^ ^56^ ^]^ Therefore, under diabetic conditions characterized by chronic inflammation and impaired immune regulation, timely inflammatory resolution while killing pathogens is critical for maintaining immune homeostasis.

To simulate the diabetic infectious microenvironment in vitro, bone marrow‐derived macrophages (BMDMs) from mice were cultured with high‐glucose and lipopolysaccharides (LPS).^[^ ^2^ ^]^ First, an 3‐(4, 5‐dimethylthiazolyl‐2)‐2, 5‐diphenyltetrazolium bromide assay was used to confirm the biocompatibility of the PTCu@GOx coating on the porous Ti disks, and the results showed that the macrophages maintained 87.29% compatibility when cultured on PTCu@GOx (Figure S16, Supporting Information). The inflammatory profile of the BMDMs treated with PTCu@GOx was evaluated using enzyme‐linked immunosorbent assay (ELISA). Results demonstrated that TNF‐α and IL‐1β levels were markedly elevated in the diabetic microenvironment. In comparison, this pro‐inflammatory trend was further exacerbated by the PTCu coating without GOx (*p* < 0.05), likely due to cuproptosis triggered by copper overload from the Cu‐CQDs, which activated copper‐dependent cell death signaling pathways. In contrast, the incorporation of GOx significantly suppressed inflammatory cytokine secretion (*p* < 0.01) and restored cytokine levels close to baseline (Figure 5B,C). ELISA analysis of IL‐10 revealed that both DM and PTCu treatment downregulated this anti‐inflammatory cytokine, whereas PTCu@GOx restored IL‐10 production (*p* < 0.05, Figure 5D), suggesting its ability to promote anti‐inflammatory phenotypic switching. Gene expression profiling further confirmed these findings: qRT‐PCR showed that pro‐inflammatory markers (*iNOS, CD86, TNF‐α*) were upregulated in DM and PTCu groups, while anti‐inflammatory markers (*CD206, Fizz1, Arg*) were suppressed. However, PTCu@GOx treatment reversed this trend, significantly downregulating pro‐inflammatory genes and enhancing anti‐inflammatory transcript levels (*p* < 0.01, Figure 5E–J). These immunomodulatory effects were corroborated by immunofluorescence staining for CD86 (M1 marker, red) and CD206 (M2 marker, green), which demonstrated a shift toward anti‐inflammatory polarization in the PTCu@GOx group (Figure 5K).

Dot blot assays were performed to quantify the expression levels of key molecules involved in inflammatory cytokine storms. Results demonstrated that PTCu@GOx significantly reduced the levels of pro‐inflammatory cytokines (granulocyte‐colony stimulating factor, IL‐1α) and chemokines (monocyte chemoattractant protein‐1) in macrophages compared to the DM group (*p* < 0.01). Additionally, the expression of TREM‐1, a critical amplifier of inflammatory responses via the PI3K/ERK pathways, was markedly downregulated (*p* < 0.05). Interestingly, IL‐2, KC, and IP‐10 levels increased in the PTCu@GOx group. Previous studies have indicated that IL‐2 enhances antigen presentation and modulates mitochondrial metabolic functions, KC suppresses hyperinflammation while promoting necrotic debris clearance, and IP‐10 regulates the autophagic clearance of dysfunctional mitochondria via p62‐mediated pathways, thereby maintaining mitochondrial integrity (Figure 5L,M; Figure S17, Supporting Information). These results suggested that the glucose‐depleting effect induced by GOx may be associated with the maintenance of mitochondrial function. Western blot analysis revealed a marked increase in AMPK phosphorylation in PTCu@GOx‐treated macrophages (Figure 5N,O). As a cellular energy sensor, AMPK is activated in response to intracellular energy fluctuations caused by GOx‐mediated glucose exhaustion.^[^ ^12^ ^]^ Accumulating evidence indicates that AMPK, which is activated during glucose starvation, alleviates the inflammatory state by orchestrating mitochondrial quality control. Specifically, phosphorylated AMPK promotes mitochondrial homeostasis by facilitating mitochondrial fission and autophagic clearance of damaged organelles and upregulating PGC‐1α to enhance mitochondrial DNA replication and respiratory chain complex expression, collectively exerting cytoprotective effects.^[^ ^12^, ^57^ ^]^ Therefore, increased phosphorylated AMPK expression could activate downstream cascades and induce cell repair processes.

To validate the differentiated therapeutic function of PTCu@GOx, simultaneous bacterial eradication, and host cell protection, a co‐culture system of *MRSA* and macrophages was established (Figure 5P). The PTCu coating induced pronounced macrophage shrinkage and apoptotic morphology, accompanied by upregulated expression of apoptosis markers (*p* < 0.01, Figure 5Q–U; Figure S18, Supporting Information). In contrast, PTCu@GOx preserved macrophage viability, as evidenced by their spread and extended physiological morphology, along with the significant downregulation of apoptotic markers (*p* < 0.01). Moreover, Western blot was carried out to detect the expression of AMPK, pAMPK, and DLAT oligomerization. The results showed that pAMPK levels were markedly downregulated in the MRSA, MRSA + PTCu, and MRSA + PTCu(+) but increased at PTCu@GOx(+). Furthermore, DLAT oligomerization—a marker of cuproptosis—was upregulated in the MRSA + PTCu and MRSA + PTCu(+) groups, but this oligomerization was significantly reduced upon GOx addition (Figure S19, Supporting Information). These results emphasized the relationship between pAMPK and cuproptosis in the co‐culture system and further indicated the protective ability of PTCu@GOx on host cells while exhibiting ideal antibacterial capability.

Current anti‐infective strategies often overlook bactericidal‐cytoprotective differentiation, leading to indiscriminate immunocyte damage and intracellular drug accumulation, which perpetuate inflammatory states. Herein, we propose a glucose exhaustion‐mediated cytoprotection mechanism. GOx depletes extracellular glucose, triggering nutrient deprivation that activates endogenous stress resistance pathways in host cells. This metabolic reprogramming not only suppresses hyperinflammation but also primes macrophages for reparative phenotypes, laying a critical foundation for subsequent tissue regeneration.

### 2.6 PTCu@GOx Protects Host Cells from Cuproptosis by Maintaining Mitochondrial Integrity

**Figure 6:**
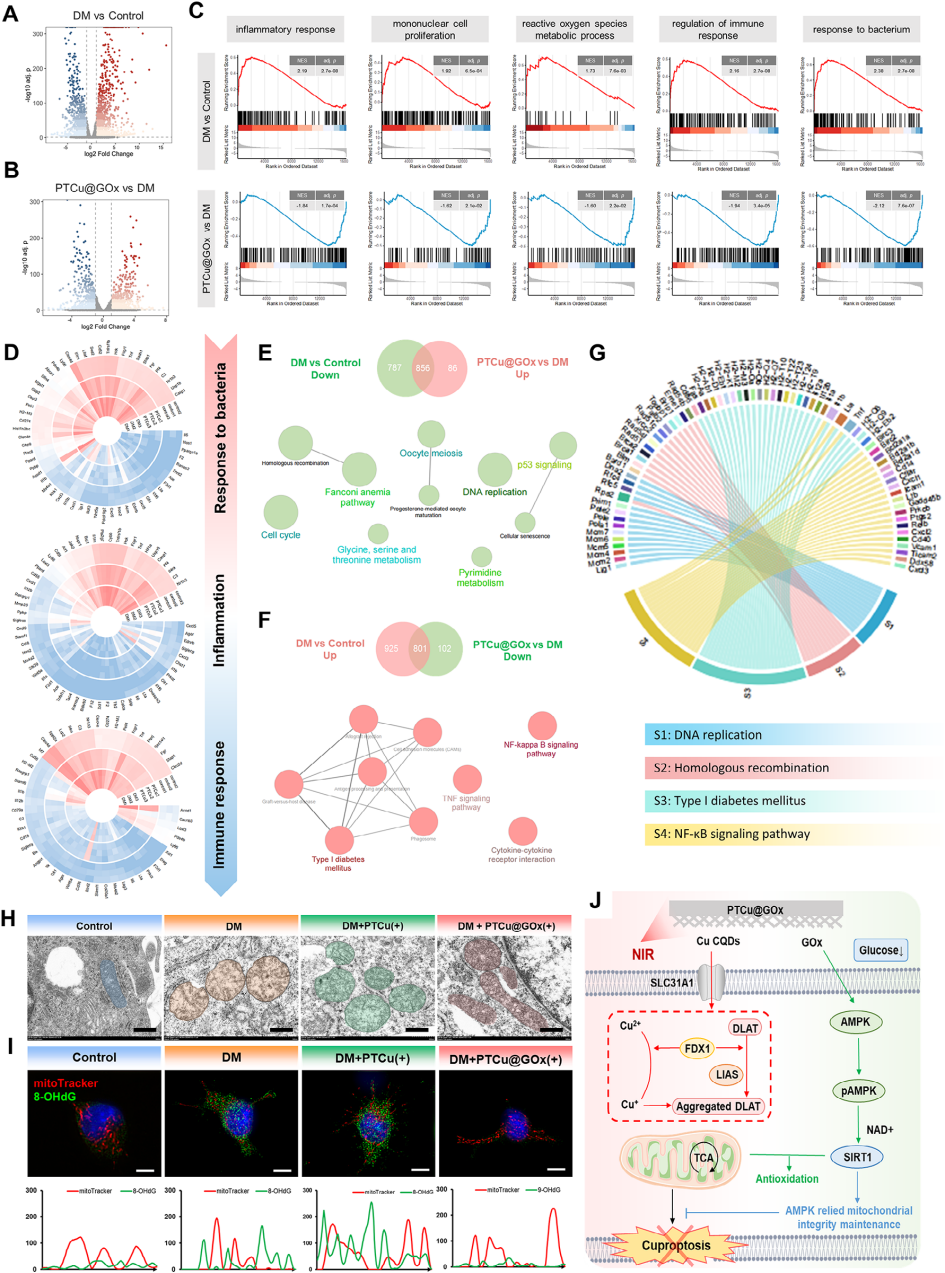
PTCu@GOx‐mediated hunger‐dependent maintenance of mitochondrial integrity and cytoprotection. A,B) Volcano plot analysis of eukaryotic transcriptome of BMDMs cultured on PTCu@GOx coated porous Ti disks; C–F) GSEA, GO, and KEGG enrichment analyses showing the differentiated expressed genes are related with mitochondrial biofunctions; G) Chord diagram of nucleic acid synthesis‐related genes and bio‐events; H) TEM analysis of mitochondrial dynamics (*n* = 3, Scale bar = 2 µm); I) mitoTracker and 8‐HOdG co‐staining showing PTCu@GOx inhibits mtDNA leakage (Scale bar = 10 µm); J) Schematic of cytoprotective mechanism of PTCu@GOx drug reservoir (*n* = 3).

To further clarify the molecular mechanisms underlying the PTCu@GOx‐mediated regulation of macrophage function under starvation‐like conditions, we performed eukaryotic transcriptome sequencing of macrophages from the control, DM, and DM+PTCu@GOx groups. Significant DEG clusters were identified between DM vs. control and DM + PTCu@GOx vs. DM. Specifically, PTCu@GOx upregulated 2,331 genes and downregulated 2,055 genes compared with those in the DM group (**Figure** **6**A,B). Among the DEGs, 408 mitochondria‐associated genes were altered, highlighting the strong link between the PTCu@GOx‐induced changes and mitochondrial regulation. Furthermore, gene set enrichment analysis revealed that DM downregulated the pathways related to the inflammatory response, ROS metabolism, and immune/infectious responses, whereas PTCu@GOx significantly reversed these trends, indicating its therapeutic potential (Figure 6C). GO and KEGG enrichment analyses further demonstrated that DEGs in the PTCu@GOx group included inflammation‐ and chemotaxis‐related genes (e.g., IL‐12 and CXCL9), which is consistent with our dot blot findings. Notably, PTCu@GOx modulated genes critical for mitochondrial dynamics (DRP1, TFAM, PGC‐1α), mtDNA transcription, and mitochondrial membrane permeability (VDAC1) (Figure 6D–F), suggesting compromised mitochondrial integrity and potential leakage of mtDNA into the cytosol, which may trigger inflammatory cascades. Additionally, the top enriched pathways further linked the DEGs to DNA replication (Figure 6G). As mtDNA is a potent pro‐inflammatory molecule, its cytosolic leakage likely activates the cGAS/STING and NLRP3 inflammasome pathways, amplifying inflammatory responses.^[^ ^58^ ^]^ Therefore, we propose that PTCu@GOx exerts cytoprotective effects by preserving mitochondrial integrity, maintaining mitochondrial dynamics and metabolic homeostasis, and suppressing mtDNA leakage to attenuate inflammation.

To corroborate these transcriptomic findings, we assessed mitochondrial ultrastructure and functionality. TEM imaging revealed that macrophages treated with DM or PTCu (without GOx) had swollen and aberrant mitochondria, whereas PTCu@GOx significantly restored mitochondrial morphology, indicating its protective effect (Figure 6H). JC‐1 staining confirmed that PTCu@GOx reversed the DM‐induced loss of the mitochondrial membrane potential and restored the red/green fluorescence ratio to near‐normal levels (Figure S20, Supporting Information). Immunofluorescence co‐staining with 8‐OHdG (an oxidized DNA marker) and MitoTracker demonstrated reduced co‐localization of damaged DNA and mitochondria in the DM and DM + PTCu groups, suggesting mtDNA oxidative damage and subsequent cytosolic leakage. In contrast, PTCu@GOx‐treated macrophages exhibited lower 8‐OHdG intensity and enhanced mitochondrial co‐localization, further supporting its role in preserving mtDNA integrity (Figure 6I). qRT‐PCR validation of mitochondrial markers further revealed that PTCu@GOx upregulated *SIRT1*, *TFAM*, and *PGC‐1α* (*p* < 0.01), indicating enhanced mitochondrial biogenesis and mtDNA stability (Figure S21, Supporting Information). Concurrently, *DRP1*, which was upregulated in the DM group and promotes mitochondrial fission and mtDNA/ROS leakage, was downregulated by PTCu@GOx (*p* < 0.01), consistent with the reduced cytosolic mtDNA leakage observed in 8‐OHdG assays. Importantly, the cuproptosis markers (*LIAS* and *FDX1*) were significantly downregulated in the PTCu@GOx group (*p* < 0.05), confirming the suppression of copper‐induced cell death. Collectively, these data demonstrate that PTCu@GOx mitigates mitochondrial dysfunction and cuproptosis, thereby shielding macrophages from the cytotoxic effects of antimicrobial agents while preserving their metabolic and inflammatory regulatory functions (Figure 6J).

### 2.7 PTCu@GOx Acts Like a “Drug Reservoir” for Ideal Long‐Term Infection and Inflammation Management in Vivo

**Figure 7:**
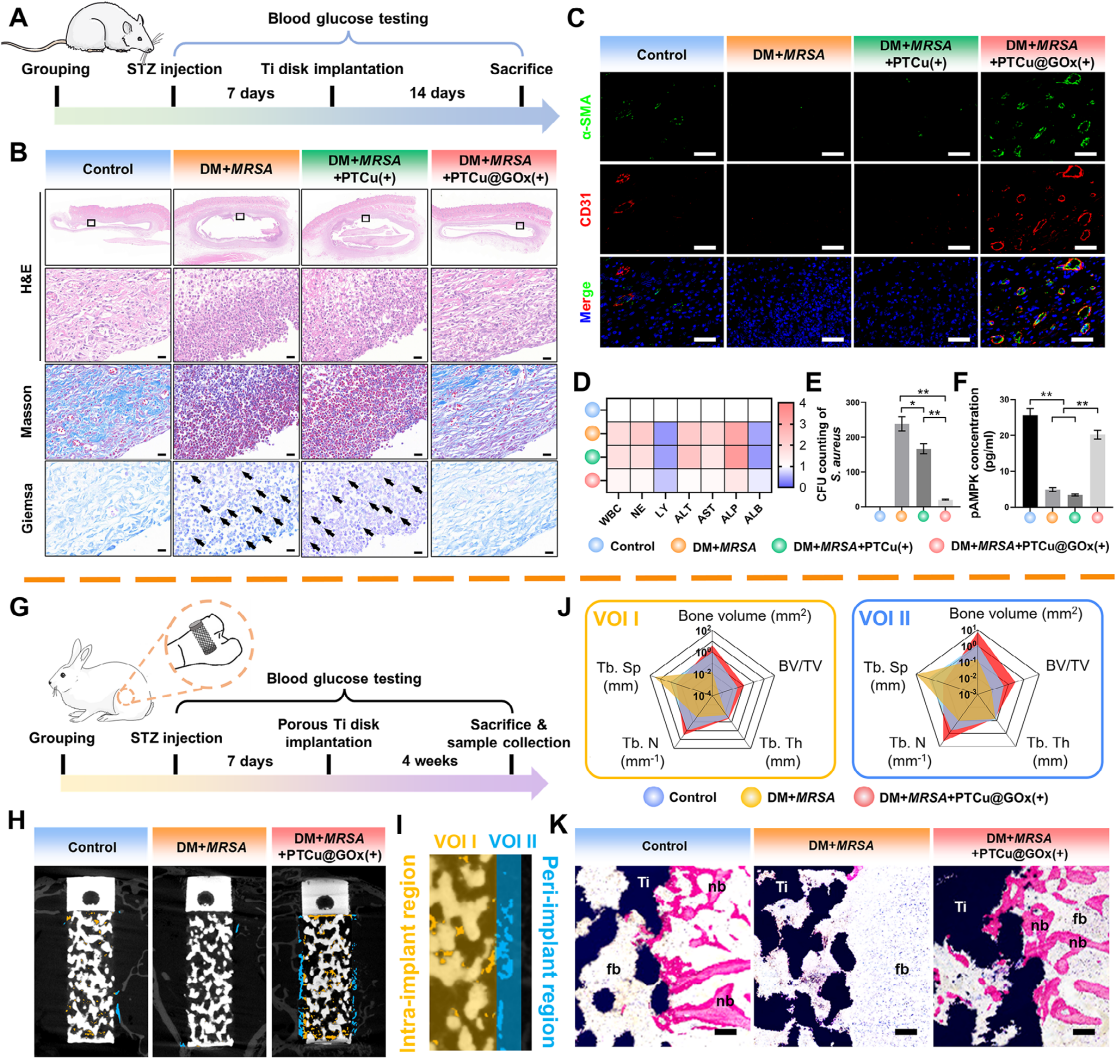
Anti‐infection and pro‐healing effects of PTCu@GOx in Sprague Dawley rats (subcutaneous) and New Zealand rabbits (femoral metaphyseal implantation models). *MRSA* suspension was injected concurrently with implantation to simulate infection conditions. A) Timeline of porous Ti disc implantation in Sprague Dawley rats (*n* = 24); B) H&E, Masson and Giemsa staining of peri‐implant soft tissues (Scale bar = 20 µm); C) α‐SMA/CD31 immunofluorescence co‐staining (Scale bar = 50 µm); D) Blood biochemical analysis post‐implantation; E) CFU counts from explanted discs at day 14; F) Tissue ELISA for pAMPK expression; G) Timeline of femoral metaphyseal defect model in rabbits (*n* = 12); H–J) MicroCT analysis of peri‐implant bone regeneration, VOI I (intra‐implant pores) and VOI II (peri‐implant) were determined for separate quantitative analysis; K) VG staining of hard tissue sections. (*p < 0.05; **p < 0.01, Scale bar = 100 µm, one‐way ANOVA with SNK post hoc test).

To validate the in vivo antibacterial efficacy of PTCu@GOx, multispecies models, including Sprague‐Dawley rats, New Zealand rabbits, and Beagle dogs, were employed for diverse implant applications. In the DM rat model of dorsal subcutaneous methicillin‐resistant *MRSA* infection, porous titanium implants were embedded at the infected sites (**Figure** **7**A). Blood biochemical analysis and hematoxylin and eosin (HE) staining of the heart, liver, spleen, lungs, and kidneys confirmed the biosafety of PTCu@GOx (Figures S22,S23, Supporting Information). Histopathological analysis revealed significant immune cell infiltration in both the DM + *MRSA* and DM + *MRSA* + PTCu(+) groups, whereas the PTCu@GOx(+) group exhibited potent bacterial eradication and accelerated inflammatory resolution (Figure 7B). Angiogenesis immunofluorescence further demonstrated enhanced tissue regeneration in the PTCu@GOx(+) group, as evidenced by the increased CD31 + vascular density (Figure 7C). The coated porous titanium implants displayed excellent biocompatibility, with minimal fibrous encapsulation and normalized inflammatory cytokine profiles in the peri‐implant tissues (Figure 7D). The bacterial CFU enumeration of tissue homogenates confirmed the superior in vivo antimicrobial activity of PTCu@GOx(+) (*p* < 0.01, Figure 7E). Notably, elevated pAMPK levels were detected by ELISA (Figure 7F), which was consistent with the macrophage western blot data, suggesting that GOx‐mediated glucose depletion activates AMPK‐dependent metabolic stress adaptation, which synergistically enhances antibacterial and tissue reparative functions.

To assess the long‐term osteogenic efficacy of the porous titanium implants serving as PTCu@GOx(+) drug reservoirs, a femoral metaphyseal defect New Zealand rabbit model was used (Figure 7G). The coating stability assesement shows that after 14‐day implantation, 62.24%±4.56% of initially loaded Cu could be maintained within the scaffold. Moreover, no significant Cu^2^⁺ accumulation in the organs were detected in the in vivo toxicity assessments (Figure S24, Supporting Information). Precisely 8 weeks after implantation, micro‐CT analysis revealed that trabecular bone‐mimetic porous titanium implants with PTCu@GOx(+) significantly enhanced de novo bone formation (Figure S25, Supporting Information). The exterior contour of the implant was demarcated to define two volumes of interest (VOI): VOI I (intra‐implant) and VOI II (peri‐implant, 500 µm outward). Semi‐quantitative analysis demonstrated an increased bone volume fraction (BV/TV) in both ROIs (*p* < 0.01), accompanied by elevated trabecular thickness (Tb.Th, Figure 7I,J). These findings were corroborated by van Gieson (VG)‐stained hard tissue sections, which exhibited denser and more contiguous neo‐bone ingrowth within and around the PTCu@GOx(+) implants (Figure 7K; Figure S21, Supporting Information). The increased osteogenesis in PTCu@GOx(+) may also results from the vascularization promotion of Cu ions. Collectively, the results indicate dual advantages of PTCu@GOx(+): early‐stage infection control through sustained Cu^2^⁺ release and long‐term tissue regeneration via AMPK‐mediated osteogenic activation. Although it is difficult to comprehensive real‐time tracking of drug release in vivo, we believe that the stability of the PTCu@GOx could be ensured by the “dimentional rise” strategy and approach a controlled drug delivery.

**Figure 8:**
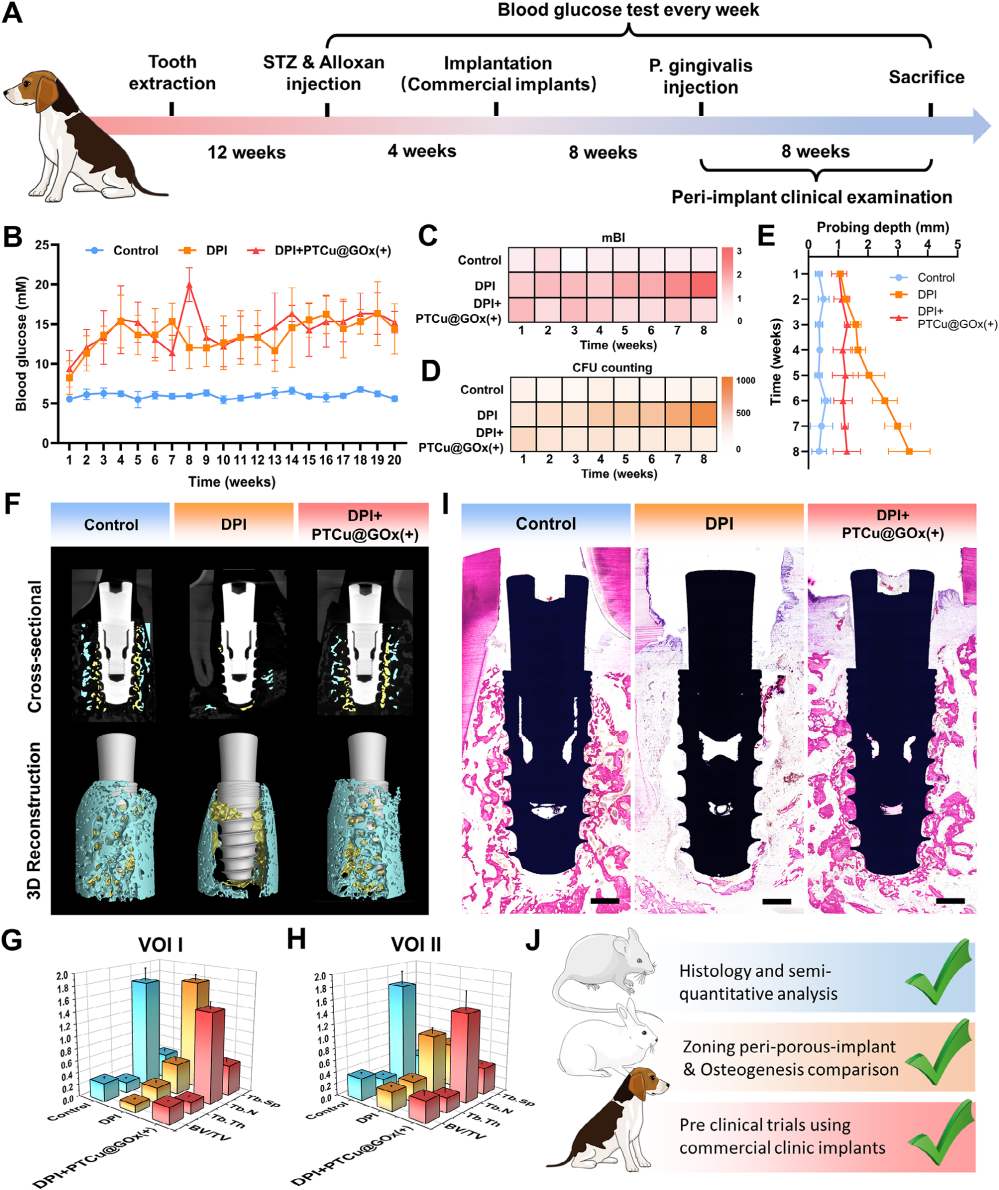
Anti‐inflammatory and pro‐healing evaluation of the PTCu@GOx drug reservoir on commercial implant surfaces in a Beagle diabetic peri‐implantitis model (*n* = 3). A) Timeline of tooth extraction, disease induction, and oral implantation in Beagles; B) Blood glucose monitoring validating diabetic status; C–E) Clinical indices of peri‐implant health including modified bleeding index, subgingival CFU counts and probing depth; F–H) High‐resolution micro‐computed tomography analysis of osteogenesis patterns, where volume of interest (VOI) I (500 µm peri‐implant) shows contact osteogenesis and VOI II (1500 µm) shows distance osteogenesis; I) van Gieson staining of hard tissue sections (Scale bar = 1 mm); J) Synergistic anti‐infection and tissue repair outcomes across rat, rabbit, and beagle models, highlighting clinical versatility and translational potential of PTCu@GOx.

Next, we evaluated the translational potential of the PTCu@GOx(+) strategy using commercial dental implants in a Beagle dog model of diabetic peri‐implantitis (DPI) (**Figure** **8**A). First, the biocompatibility of the coating on commercial implants in a large animal model was confirmed by HE staining of the heart, liver, spleen, lungs, and kidneys (Figure S26, Supporting Information). Following diabetes induction via STZ and alloxan application, sustained hyperglycemia (blood glucose levels >11.1 mm) confirmed successful diabetes modeling (Figure 8B). A modified probe bleeding index revealed progressive bleeding aggravation in the DPI group, whereas PTCu@GOx(+) significantly ameliorated inflammatory bleeding.^[^ ^59^ ^]^ The bacterial culture of the peri‐implant sulcular fluid demonstrated escalating infection severity in the DPI group over time, in contrast to the markedly reduced microbial loads in the PTCu@GOx(+) group (Figure 8C,D). Additionally, the probing depth, a key indicator of peri‐implant tissue loss, increased to 3.38 ± 0.69 mm in the DPI group but remained stable at 1.29 ± 0.46 mm in the PTCu@GOx(+) group at 8 weeks post‐modeling (Figure 8E), underscoring its capacity to inhibit tissue degradation. High‐resolution micro‐CT also revealed substantial bone loss around the DPI‐treated implants, whereas PTCu@GOx(+) significantly enhanced the peri‐implant bone volume. Quantification of osteogenic parameters within two VOIs, 500 µm (contact osteogenesis) and 1500 µm (distant osteogenesis) from the implant surface, demonstrated that the BV/TV, Tb.Th, and trabecular number were significantly increased (*p* < 0.01, Figure 8F–H). VG staining showed that the PTCu@GOx coating on commercial dental implants boosted new bone formation. This was further confirmed by the bone‐implant‐contact (BIC) interface evaluation, which showed a significant increase in BIC in the PTCu@GOx group compared to that in the DPI group (Figure 8I; Figure S27, Supporting Information).

In summary, our study demonstrated the dual efficacy of PTCu@GOx in combating infection and enhancing cytoprotective/tissue‐regenerative functions under diabetic conditions, which was validated using 3D‐printed and commercial implants in multiple preclinical animal models. These findings highlight its translational promise as a versatile therapeutic platform that bridges antimicrobial performance with metabolic reprogramming to address the multidimensional challenges of diabetic infection. This study establishes a paradigm for precision‐engineered implants targeting both pathogen eradication and host microenvironment modulation, paving the way for clinical translation in diabetes‐associated complications.

## 3 Conclusion

In this study, we successfully engineered a trabecular bone‐mimetic 3D‐printed porous titanium implant based on dimensional rising theory, followed by the construction of micro‐nano hierarchical surface structures to amplify the effective surface area for functional coating deposition. We first evaluated the tribological resistance of dimensionally raised Ti implants functioning as 3D drug reservoirs, which is critical for retaining therapeutic payloads during clinical implantation scenarios. By incorporating Cu‐CQDs, the implants were endowed with bioregulatory activity and exhibited an ideal capacity for infection management and tissue regeneration in diabetic bone defects. Under NIR stimulation, the photothermal effect disrupted bacterial phospholipid bilayers, enhancing Cu^2^⁺ internalization to induce metabolic interference and cuproptosis‐like cell death via lipid peroxidation.

Furthermore, intracellular Cu^2^⁺ accumulation in host cells was counterbalanced by GOx‐mediated glucose depletion, which triggered AMPK phosphorylation and SIRT1 overexpression to activate mitochondrial integrity maintenance pathways. This metabolic reprogramming attenuated the macrophage inflammatory responses while promoting cytoprotective adaptation. Validated across multiple animal models, the PTCu@GOx strategy demonstrated therapeutic versatility and clinical translation potential for different implantation patterns. Collectively, this study establishes a differentiated intervention strategy that simultaneously targets pathogens via cuproptosis and safeguards host cells through metabolic modulation, offering a promising therapeutic strategy for repairing infectious tissue defects in patients with diabetes.

## 4 Experimental Section

### 1st Dimensional Rise: Preparation of Titanium Plate and Porous Titanium Implant

A cylindrical structure (ϕ = 3 mm × 8 mm) was digitally designed with biomimetic trabecular porosity using computer‐aided design and printed via selective laser melting using grade 4 commercially pure titanium powder. The detailed printing marameter are described in Supporting Information. The implants exhibited an average pore size of 385 ± 1.38 µm, trabecular strut diameter of 200 ± 0.67 µm, and porosity of 76.82% ± 0.83%.

### Synthesis of Copper‐Doped Luteolin Carbon Quantum Dots

The Cu‐CQDs were synthesized via a one‐pot hydrothermal method. Briefly, 0.1 m CuSO₄·5H₂O and 50 mg luteolin were dissolved in 20 mL formamide under vigorous stirring for 30 min and heated at 160 °C for 8 h. anhydrous ethanol and centrifugation were used for purification. Detailed method is described in Supporting Information.

### 2nd Dimensional Rise: Surface Micro/Nano Hierackical Surface Modification and CQDs Loading

The micro/nano hierarchical structures on titanium surfaces were fabricated via alkali‐thermal treatment based on our previously study.^[^ ^29^ ^]^ Detailed method is described in Supporting Information.

### Physical and Chemical Characterization

The characteristics of Cu‐CQDs and PTCu@GOx coatings were tested using TEM, XPS, UV–vis, ESR Spectroscopy etc. Detailed method is described in Supporting Information.

### Cell Culture and in Vitro Diabetic Infection Model Creation

RAW264.7 and bone marrow‐derived macrophages were cultured in DMEM supplemented with 10% FBS and 1% penicillin‐streptomycin at 37 °C under 5% CO₂. To mimic diabetic infectious conditions, the medium was further supplemented with additional glucose and LPS. For co‐culture experiments, cells were seeded onto sterilized Ti disks (1 × 10⁴ cells cm^−^^2^) and allowed to adhere for 12 h. *MRSA* (ATCC 43300) suspensions (1 × 10⁶ CFU mL^−1^) were then added to the cell culture system and incubated for 24 h. The grouping of macrophages were Control, DM, DM + PTCu, DM + PTCu(+) (with NIR), DM + PTCu@GOx(+) (with NIR). Detailed in vitro evaluation is described in Supporting Information.

### In Vivo Analysis

All the in vivo experiments had complied with the ARRIVE guidelines and had been approved (CQHS‐REC‐2020(LSNo.75)) by the Ethics Committee of School of Stomatology, Chongqing Medical University. Detailed in vivo evaluation is described in Supporting Information.

### Statistical Analysis

All data in this study were analyzed using SPSS 23.0 (IBM, USA). For multi‐group comparisons, one‐way ANOVA with the Student‐Newman‐Keuls post hoc test was applied. The data was presented as mean ±SD. For quantitative analyses, the sample size was at least *n* = 3. Significance was defined as *p < 0.05 and ​**​*p* < 0.01.

## Conflict of Interest

The authors declare no conflict of interest.

## Supporting information



Supporting information

## Data Availability

The data that support the findings of this study are available in the supplementary material of this article.
