# Supplementary material for: Volumetric Engineered 3D Drug Reservoir Against Diabetic Implant Infection via Cuproptosis‐Like Bacterial Death and Hunger‐Triggered Maintenance of Mitochondrial Integrity
Source: Adv Sci (Weinh). 2025 Jul 3;12(37):e06554. doi: 10.1002/advs.202506554 (PMC12499384; doi:10.1002/advs.202506554)
Supplement: Supplementary file 1 — Supporting information [file ADVS-12-e06554-s001.docx]

**Supporting Information**

**Volumetric engineered 3D drug reservoir against diabetic implant infection via cuproptosis-like bacterial death and hunger-triggered maintenance of mitochondrial integrity**

Dize Li^1,3^, Hongrui Qin^1^, Min Jiang^1^, Hongjiang Wei^1^, Hongyong Zhao^1^, Ping Tang^1,2^, Guangyu Jian^1^, Henny C van der Mei^3^, Tao Chen^1,∗^

^1^Stomatological Hospital of Chongqing Medical University, Chongqing Key Laboratory of Oral Diseases and Biomedical Sciences, Chongqing Municipal Key Laboratory of Oral Biomedical Engineering of Higher Education, Chongqing Medical University, Chongqing, 401147, P. R. China

^2^The Third Hospital of Mianyang, Sichuan Mental Health Center, Mianyang, 621000, P. R. China

^3^University of Groningen and University Medical Center Groningen, Department of Biomaterials & Biomedical Technology, Hanzeplein 1, Groningen, 9613 GZ, The Netherlands

^∗^Corresponding author：chentao1985@hospital.cqmu.edu.cn

**Methods**

**1^st^ dimensional rise: Preparation of Titanium Plate and Porous Titanium Implant**

A cylindrical structure (ϕ = 3 mm × 8 mm) was digitally designed with biomimetic trabecular porosity using computer-aided design and printed via selective laser melting using grade 4 commercially pure titanium powder. The printing parameters are as follows: laser power​​: 200 W; wavelength​​: 1,075 nm; spot diameter​​: 50–80 µm; layer thickness​​: 20–50 μm. The post-processing steps​​ include: stress relief​​: annealed at 650°C for 2 hours under argon; ultrasonic cleaning​​: 30 min in acetone, followed by ethanol. The implants exhibited an average pore size of 385 ± 1.38 μm, trabecular strut diameter of 200 ± 0.67 μm, and porosity of 76.82 ± 0.83%.

**Synthesis of Copper-doped Luteolin Carbon Quantum Dots**

The copper-doped carbon quantum dots (Cu-CQDs) were synthesized via a one-pot hydrothermal method. Briefly, 0.1 M copper sulfate (CuSO₄·5H₂O) and 50 mg luteolin were dissolved in 20 mL formamide under vigorous stirring for 30 min. The homogeneous mixture was transferred into a 50 mL Teflon-lined stainless-steel autoclave and heated at 160°C for 8 h. After cooling to room temperature, the resulting dark-brown solution was mixed with 100 mL anhydrous ethanol and concentrated via rotary evaporation at 60°C to remove residual solvents. The crude product was further purified by centrifugation (12,000 rpm, 20 min) to eliminate unreacted precursors and large aggregates. Finally, the purified Cu-CQDs were obtained as a solid powder after freeze-drying (-50°C, 24 h) for subsequent characterization and application.

**2^nd^ dimensional rise: Surface Micro/Nano Hierackical Surface Modification and CQDs loading**

The micro/nano hierarchical structures on titanium surfaces were fabricated via alkali-thermal treatment. Briefly, Ti disks were sequentially cleaned with deionized water, dried, and heated in a muffle furnace under an air atmosphere. The temperature was ramped at 5°C/min to 600°C, held for 1 h, and then allowed to cool naturally. For the PDA-TSPBA coating, the sintered Ti disks were immersed in a freshly prepared solution containing 2 mg/mL dopamine hydrochloride and 1 mM tetra-arm phenylboronic acid (TSPBA) in Tris-HCl buffer (pH 8.5). The mixture was incubated at room temperature for 6 h under gentle agitation (50 rpm) to facilitate co-deposition of PDA and TSPBA. Post-reaction, the coated Ti disks were thoroughly rinsed with deionized water for 30 min and air-dried. Moreover, to immobilize the CQDs onto 3D-printed trabecular bone-mimetic porous titanium implants, the purified CQD solution was combined with the titanium substrates under ultrasonication for 30 min to enhance penetration into the porous architecture. The substrates were then incubated in the solution at 37°C for 24 h to promote CQD adhesion. Finally, the CQDs-loaded titanium implants were vacuum-dried at 60°C for 12 h to ensure stable coating formation.

**TEM analysis**

To prepare the samples for TEM measurements, Cu-CQDs were dispersed into ethanol and then dripped the suspensions on formvar stabilized with carbon support film (Formvar/Carbon 230 mesh) followed by drying in air.

**DLS measurement**

The average diameter of Cu-CQDs was measured using a Zetasizer® Nano-S (NanoBrook 90Plus PALS) at 25 °C by dispersion of nanoparticles in HEPES buffer (5 mM) with adjusted pH (using HCl or NaOH).

**Scanning Electron Microscope and Energy-dispersive X-ray Spectroscopy Mapping**

The microstructures of the PTCu@GOx coated Ti disks were observed by scanning electron microscopy (FEI, Quanta 450, USA). Before imaging, all samples were ultrasonically cleaned in ethanol (99.7%) for 10 min and dried under nitrogen flow to eliminate surface contaminants. To enhance conductivity and mitigate charging effects, the specimens were sputter-coated with a 5 nm Au/Pd layer using a Gatan PECS™ 682 precision etching coating system (15 mA, 60 s deposition time). SEM observations were performed at an accelerating voltage of 5 kV and a working distance of 8–10 mm. Secondary electron and backscattered electron signals were simultaneously collected to resolve both topographical features and elemental contrast of the PTCu@GOx coating. Following SEM imaging, energy-dispersive X-ray spectroscopy (EDS) mapping was conducted using an Oxford Instruments X-MaxN 80 mm² silicon drift detector integrated with the FEI Quanta 450 system. The SEM mode was switched to high-vacuum mode (<10⁻³ Pa) to minimize air scattering effects for EDS. Then the elemental mappings for Cu, C, O, N, and Ti were generated using AztecEnergy software.

**XPS Characterization Methodology**

X-ray photoelectron spectroscopy (XPS) analysis was performed on both CQDs and PTCu@GOx/Ti using Thermo Scientific K-Alpha+ spectrometer equipped with a monochromatic Al Kα X-ray source (1,486.6 eV). Prior to analysis, samples were ultrasonically cleaned in ethanol for 5 min and dried under a nitrogen stream to remove surface contaminants. To minimize charging effects, a combined electron flood gun and low-energy Ar⁺ ion beam (10 eV, 10 s) was employed for charge neutralization. Survey scans were acquired over a binding energy range of 0–1,200 eV with a pass energy of 200 eV and a step size of 1.0 eV. High-resolution spectra for C 1s, O 1s, Ti 2p, Cu 2p, and N 1s were collected at a pass energy of 50 eV and a step size of 0.1 eV.

**Fourier Transform Infrared (FTIR) Spectroscopy**

FTIR spectra of the Ti-OH substrate, PDA, TSPBA, Cu-CQDs, and PTCu@GOx composite coating were acquired using a Thermo Scientific Nicolet iS50 FTIR spectrometer equipped with a diamond attenuated total reflectance (ATR) accessory. Spectra were recorded over a wavenumber range of 400–4,000 cm⁻¹. Triplicate measurements were performed for each sample to ensure reproducibility, and spectral shifts >5 cm⁻¹ were flagged for reanalysis.

**Water Contact Angle Measurement and Protein Adsorption**

The hydrophilic/hydrophobic properties of uncoated Ti disks and PTCu@GOx-coated Ti disks were evaluated via static water contact angle measurements using a DataPhysics OCA 25 contact angle goniometer equipped with a high-speed CCD camera. Prior to testing, all samples were ultrasonically cleaned in ethanol (99.7%) for 10 min and dried under a nitrogen stream to remove surface contaminants. Ultrapure water droplets (3 μL) were dispensed onto the sample surface at a controlled injection rate of 0.5 μL/s using a precision microsyringe. The droplet profile was captured within 5 s of deposition. Quantifiably, a Micro BCA Protein Assay Reagent Kit (Thermo Scientific, USA) was applied to measure absorbance at 570 nm on a Multimode Plate Reader (PerkinElmer, EnSpire, USA) to test the protein adsorption property of PTCu@GOx coating.

**ESR Spectroscopy for ROS Detection**

The generation of hydroxyl radicals (•OH), singlet oxygen (¹O₂), and superoxide anions (O₂•⁻) by the PTCu@GOx coating under NIR irradiation was investigated using a Bruker EMXplus X-band ESR spectrometer equipped with a high-sensitivity cavity (ER 4122SHQE). All measurements were conducted at 25°C, and time-sweep spectra were acquired over 10 min post-irradiation to monitor ROS dynamics. ESR signals were analyzed using Bruker Xenon software.

**Fluorescence and Phosphorescence Spectroscopy Measurement**

The photoluminescence properties were characterized using a fluorescence spectrophotometer (PerkinElmer). Emission spectra were recorded in the range of 350–700 nm with an excitation wavelength set at 360 nm. Slit widths for excitation and emission were fixed at 5 nm, and scan speed was 240 nm/min. For phosphorescence measurements, the same instrument was operated in time-resolved mode with a pulsed xenon lamp. All spectra were corrected for instrument response and background solvent signals.

**UV-Vis Absorption Spectroscopy**

UV-Vis absorption spectra were recorded using a double-beam spectrophotometer (Shimadzu) at room temperature. The absorbance was measured over a wavelength range of 200–800 nm with a scan speed of 400 nm/min and a slit width of 2 nm.

**Photothermal Performance Evaluation**

The photothermal response of PTCu@GOx-coated porous titanium implants was systematically evaluated under varying NIR laser currents (1250, 1350, and 1450 mA) using an FLIR T860 infrared thermal camera with a thermal sensitivity of ≤0.03°C. The 808 nm NIR laser (spot diameter: 5 mm, power density calibrated to 1.0, 1.5, and 2.0 W/cm², respectively) was vertically aligned to irradiate the sample surface at a fixed distance of 10 cm. Prior to testing, all samples were equilibrated to ambient temperature (25 ± 0.5°C). Thermal images were captured at 1-, 3-, and 5-min intervals post-irradiation, with real-time temperature profiles extracted using FLIR Research Studio Max software. Temperature data were averaged across three independent replicates (n = 3) and normalized to baseline (ΔT = Tt – T0). The photothermal conversion efficiency (η) was calculated via the Roper model:

$$\eta=\frac{hA\Delta T-Q_{loss}}{I(1-{10}^{-A})}\times100\%$$

where h is the heat transfer coefficient (12.5 mW/cm²·°C), A the sample absorbance (measured via UV-Vis-NIR spectroscopy), Qloss the ambient heat dissipation (determined from control experiments), and I the incident laser power.

Furthermore, photothermal stability of the PTCu@GOx coating was evaluated using an switch on/off cycling method. An 808 nm laser (1.5 W/cm²) was alternately switched on for 10 min and off for 10 min over four consecutive cycles (total duration: 80 min). Real-time temperature monitoring was performed using infrared thermal camera with a sampling interval of 30 s.

**Copper Ion Release Profiling**

To assess the sustained Cu²⁺ release kinetics, PTCu@GOx-coated and uncoated porous/smooth titanium disks (n = 5) were immersed in 2 mL of phosphate-buffered saline (PBS, pH 7.4) and incubated on an orbital shaker at 25°C. Daily, 1 mL of supernatant was collected and replaced with an equal volume of fresh PBS to maintain the total volume of liquid over 14 days. Collected aliquots were centrifuged (10,000 rpm, 10 min) to remove particulates and stored at −80°C until analysis. Copper ion concentrations were quantified using inductively coupled plasma optical emission spectrometry (ICP-OES) with the plasma power of 1.2 kW and integration time of 5 s.

**Abrasion Resistance and Sustained Drug Release Assessment**

To evaluate the tribological durability and drug retention capacity of the PTCu@GOx coating, both coated porous and smooth titanium disks (n = 5) were subjected to abrasion using 400-grit silicon carbide sandpaper under a constant normal force of 500 N for 10 cycles (1 cycle = 10 cm linear abrasion). After that, samples were immersed in 2 mL of phosphate-buffered saline (PBS, pH 7.4) at 25°C. 1 ml of supernatant was collected after 3 days and analyzed via ICP-OES to quantify Cu²⁺ release.

**Antibacterial assay**

Gram-negative Escherichia coli (*E. coli*, ATCC 25922), Gram-positive Staphylococcus aureus (*S. aureus*, ATCC 25923), and methicillin-resistant *S. aureus* (MRSA, ATCC 43300) were selected for antimicrobial assays. Bacterial suspensions were prepared in tryptic soy broth (TSB) and brian heart infusion (BHI) to a final concentration of 1 × 10⁶ CFU/mL, verified via optical density (OD600) and colony counting. For leachable antibiosis, coated and uncoated Ti disks were immersed in bacterial suspensions (2 mL per disk) and incubated statically at 37°C for 24 h. Post-incubation, 100 μL aliquots were serially diluted, plated on agar, and incubated for 24 h at 37°C to enumerate surviving colonies. Nucleic acid release (OD260/280) and protein leakage (BCA assay) were quantified to assess bacterial membrane disruption. For biofilm evaluation, Ti disks were co-cultured with bacterial suspensions (1 × 10⁶ CFU/mL) for 48 h under static conditions. Biofilms were stained using SYTO 9 (live, green fluorescence) and propidium iodide (dead, red fluorescence) (LIVE/DEAD® BacLight™ Kit). A Leica TCS SP8 confocal laser scanning microscope (CLSM) captured z-stack images (1 μm step size), and biofilm biomass/thickness were reconstructed using Imaris v9.7. Besides, bacterial suspensions were incubated with alamar blue dye for 2 h at 37°C. Fluorescence intensity (ex/em: 555/590 nm) was measured using microplate reader.

After 12 h of co-culture with bacterial suspensions (1 × 10⁶ CFU/mL), the titanium samples were gently rinsed three times with PBS to remove non-adherent bacteria. Biofilms were fixed with 2.5% glutaraldehyde in 0.1 M cacodylate buffer for 2 h at 4°C, followed by dehydration through a graded ethanol series. Critical point drying was performed using a Leica EM CPD300 with liquid CO₂ as the transition fluid. Then, bacteria were observed through FEI Quanta 450 FEG-SEM at an accelerating voltage of 5 kV.

**Inhibition Zone Assay**

Bacterial suspensions of *S. aureus* and *E. coli* (1 × 10⁶ CFU/mL) were spread on agar plates. Then the PTCu@GOx coated and uncoated porous Ti disks were placed on the agar surface. After 24 h incubation at 37°C in the incubator, the inhibition zones surrounding the disks were photographed and the area was quantified using ImageJ software.

**Molecular Dynamics and Docking**

Molecular docking between peptidoglycan and TSPBA was performed using AutoDock Vina, with TSPBA parametrized via the GAFF force field. MD simulations were conducted in GROMACS: the complex was solvated in a 10 × 10 × 10 nm³ TIP3P water box (1.2 nm buffer distance), neutralized with ions, and energy-minimized (50,000 steepest descent steps). Systems were equilibrated under NVT (300 K, V-rescale) and NPT (1 bar, Berendsen) ensembles, followed by a 100 ns production run with periodic boundary conditions. Electrostatic and van der Waals interactions were calculated using PME and a 10 Å cutoff, respectively. Besides,trajectories were analyzed for RMSD, radius of gyration (Rg), and hydrogen bonding using GROMACS utilities, visualized in PyMOL/VMD.

In order to observe that the PTT helps increase the permeability of cell membrane of bacteria, we then used molecular dynamics simulation to show the changes of phospholipid bilayer. Specifically, the topology and configuration files for lipid bilayers at 25°C (298 K) and 50°C (323 K) were generated using CHARMM-GUI. Each bilayer comprised 60% POPG, 15% POPE, 15% DOTAP, and 10% TOCL, with 160 lipid molecules per leaflet (320 total), solvated in a 10 × 10 × 10 nm³ periodic box containing 17.5 Å water layers (32,133 atoms total). The system was neutralized with 234 Na⁺ and 26 Cl⁻ ions and simulated using GROMACS 2023 with the CHARMM36m force field for lipids and TIP3P water model, maintaining a minimum 1.2 nm buffer distance between the bilayer edge and box boundaries. Moreover, simulations followed the CHARMM-GUI workflow: energy minimization via steepest descent (5,000 steps), 1,875 ps of pre-equilibration with positional restraints on lipids, and a 100 ns production run under the NPT ensemble. Electrostatic interactions were treated with PME, and nonbonded interaction cutoffs were set to 10 Å. Temperature (300 K) and pressure (1 bar, semiisotropic) were regulated using the Nose-Hoover thermostat and Parrinello-Rahman barostat, respectively. All bonds were constrained via the LINCS algorithm.

**Intracellular Copper Accumulation of Bacteria**

To evaluate the intracellular copper accumulation in S. aureus following co-culture with PTCu@GOx-coated Ti disks, bacterial cells were harvested and processed as follows: After 24 h of co-culture in TSB medium (1 × 10⁶ CFU/mL), the bacterial suspension was centrifuged at 8,000 rpm for 10 min to pellet cells. The pellet was washed thrice with PBS (pH 7.4) to remove extracellular Cu²⁺ ions and resuspended in 1 mL lysis buffer (10 mM Tris-HCl, 1% SDS, protease inhibitors). Bacterial cells were lysed via ultrasonication on ice. The lysate was centrifuged at 12,000 rpm for 15 min to remove debris, and the supernatant was analyzed via ICP-OES.

**Transcriptomic Profiling of MRSA via RNA-Seq**

**1. RNA Quality Control and Library Preparation**

Total RNA was extracted from MRSA co-cultured with PTCu@GOx-coated titanium disks for 12 h. RNA integrity was assessed via 1% agarose gel electrophoresis to monitor degradation/contamination and further quantified using the Agilent Bioanalyzer 2100 system (RNA Nano 6000 Assay Kit). Ribosomal RNA depletion was performed via ethanol precipitation, followed by cDNA synthesis with random hexamer primers and dUTP incorporation for strand-specific library construction. Libraries underwent end repair, A-tailing, adapter ligation, size selection (250–300 bp), USER enzyme digestion, and PCR amplification. Final libraries were quantified using Qubit Fluorometry and validated for size distribution via bioanalyzer.

**2. Sequencing and Bioinformatics Analysis**

Indexed libraries were clustered on an Illumina cBot Cluster Generation System (TruSeq PE Cluster Kit v3-cBot-HS) and sequenced on an Illumina NovaSeq platform (150 bp paired-end reads). Raw reads were processed through fastp to remove adapters, poly-N sequences, and low-quality reads (Q20/Q30 >90%), generating high-quality clean data. Clean reads were aligned to the *Staphylococcus aureus* reference genome (NCTC 8325, downloaded from NCBI) using Bowtie2 v2.2.3.

**3. Transcriptome Annotation and Differential Expression**

Novel genes, operons, transcription start/termination sites (TSS/TTS), and antisense transcripts were identified via Rockhopper. Promoter regions (700 bp upstream of TSS) were predicted using a Time-Delay Neural Network (TDNN). Untranslated regions (UTRs), Shine-Dalgarno (SD) sequences, and terminators were annotated using RBSfinder and TransTermH. Small RNAs (sRNAs) were predicted by blasting novel intergenic transcripts against the nr database, with secondary structures and targets analyzed via RNAfold and IntaRNA.

Gene expression levels were quantified as FPKM (Fragments Per Kilobase Million) using HTSeq v0.6.1. Differential expression analysis (PTCu@GOx vs. control) was performed with DESeq v1.18.0 (for biological replicates) or DEGSeq v1.20.0 (without replicates), applying thresholds of |log2 fold change| >1 and FDR-adjusted *p* <0.05 (Benjamini-Hochberg correction). Enriched Gene Ontology (GO) terms and KEGG pathways were identified via GOseq (correcting for gene length bias) and KOBAS, respectively, with significance set at *p* <0.05.

**qRT-PCR Analysis**

Total RNA was extracted from *S. aureus* using TRIzol® reagent (Invitrogen), followed by DNase I treatment (Thermo Scientific) to eliminate genomic DNA. First-strand cDNA was synthesized using the PrimeScript™ RT Reagent Kit (Takara Bio) following the instruction of manufacturer. qRT-PCR was performed in triplicate using TB Green® Premix Ex Taq™ II (Takara Bio) on a Bio-Rad CFX96 Real-Time System under the following conditions: 95°C for 30 s, 40 cycles of 95°C for 5 s, and 60°C for 30 s. Melt curve analysis (65–95°C, 0.5°C increments) confirmed primer specificity.

Primer sequences we used were as follows: *sufB*, 5’GCTGAAGCAATTGACGAACG3’ and 5’TTGCTTGTTGCGTTTCTTTG3’; *sufC*, 5’ATGGCAGAAAAACGCATTGAC3’ and 5’TCAGCGTTGCTTGTTGCGTTT3’; *DLAT*, 5’GCAGCAGTATCATTAGAAGGCGA3’ and 5’TTCTTACGGTGATCCCATAATGC3’; *GPX4*, 5’TGCGTAAATAACGGATGTTCATC3’ and 5’ATCGTGGGTTTGTAGTGTTGAGT3’; *FDX1*, 5’ATGGATACTTGTATTGCATGTGGTG3’ and 5’TAAAGCGTCCCCATCAAATGAT3’; *LIAS*, 5’ACCACTTAAATCGGGTCAAGAAC3’ and 5’GAATGCTTGTTTAGCGATGGA3’; 16S rRNA, 5’ACAATACAAAGGGCAGCGAAAC3’ and 5’CGATACGGCTACCTTGTTACGAC3’.

**Fluorescence Staining of Different Type of ROS**

The intracellular ROS levels in *S. aureus* and *E. coli* cultured on PTCu@GOx-coated porous titanium implants were quantified using fluorogenic probes targeting distinct ROS species. Bacterial suspensions (1 × 10^6^ CFU/mL) were incubated with the following dyes for 30 min at 37°C in the dark:

- **HKPerox-2** (10 μM, H₂O₂-specific, Ex/Em = 488/525 nm),
- **Dihydroethidium (DHE)** (5 μM, O₂⁻•-specific, Ex/Em = 518/605 nm),
- **DAF-FM DA** (5 μM, NO-specific, Ex/Em = 495/515 nm),
- **DCFH-DA** (10 μM, general ROS, Ex/Em = 485/535 nm).

Post-incubation, cells were washed thrice with PBS (pH 7.4) to remove unbound probes. Fluorescence intensity was measured using a Leica TCS SP8 confocal microscope.

**ATP Assay**

ATP levels were quantified using an ATP Assay Kit (Beyotime Biotechnology, China). Briefly, *S. aureus*, *E. coli*, and *P. gingivalis* were inoculated onto porous titanium scaffolds ​​with or without PTCu@GOx coating​​ and exposed to ​​NIR irradiation​​ (808 nm, 1.5 W/cm²) for 30 min, followed by 12 h of co-culture. Bacterial pellets were collected by centrifugation (8,000 ×g, 10 min), lysed with the provided buffer, and processed according to the manufacturer’s protocol. ATP concentrations in the lysates were measured using a microplate reader at 560 nm.

**Malondialdehyde Assay**

Lipid peroxidation in *S. aureus* and *E. coli* cultured on PTCu@GOx-coated porous titanium implants were measured using the Lipid Peroxidation MDA Assay Kit (Beyotime, China) following the manufacturer’s protocol. Bacterial suspensions (1 × 10^6^ CFU/mL) were co-cultured with the coated implants for 12 h, harvested via centrifugation (8,000 ×g, 10 min), and lysed in RIPA buffer containing protease inhibitors. Lysates were centrifuged at 12,000 ×g for 15 min to remove debris. The supernatant (100 μL) was reacted with 200 μL of TBA working solution at 95°C for 60 min. After cooling on ice, samples were centrifuged (10,000 ×g, 10 min), and the absorbance of the supernatant was measured at 532 nm using microplate reader.

**TUNEL Assay**

Bacterial apoptosis in *S. aureus* and *E. coli* was evaluated using the One-Step TUNEL Apoptosis Assay Kit (Beyotime, China). Bacterial suspensions (1 × 10^6^ CFU/mL) were fixed with 4% paraformaldehyde for 30 min, permeabilized with 0.1% Triton X-100 for 15 min, and labeled with TUNEL reaction mixture (50 μL) at 37°C for 60 min in the dark. After washing with PBS (pH 7.4), nuclei were counterstained with DAPI (1 μg/mL). After that, images were acquired using a Leica TCS SP8 confocal microscope (63× oil immersion objective). The 3D Surface Plot was used to semi-quantify TUNEL-positive signals.

**Cell Culture and *in vitro* Diabetic Infection Model Creation**

RAW264.7 and bone marrow-derived macrophages (BMDMs) were cultured in DMEM supplemented with 10% fetal bovine serum (FBS) and 1% penicillin-streptomycin at 37°C under 5% CO₂. To mimic diabetic infectious conditions, the medium was further supplemented with additional glucose (25 mM) and LPS (100 ng/mL). For co-culture experiments, cells were seeded onto sterilized Ti disks (1 × 10⁴ cells/cm²) and allowed to adhere for 12 h. MRSA (ATCC 43300) suspensions (1 × 10⁶ CFU/mL) were then added to the cell culture system and incubated for 24 h. The grouping of macrophages were Control, DM (high glucose + LPS), DM+PTCu, DM+PTCu(+) (with NIR), DM+PTCu@GOx(+) (with NIR).

**MTT Assay**

After culturing macrophages on the coated porous Ti disks for 24 h, MTT agent (Beyotime, China) was used and incubated for 4 h at 37°C. Then, the optical density was measured at 570 nm using microplate reader.

**ELISA Analysis**

Supernatants from macrophages were collected at 24 h post-treatment. The concentrations of TNF-α, IL-1β, and IL-10 were quantified using commercial ELISA kits (R&D, USA) following the manufacturer’s protocol. Briefly, 100 μL of supernatant or serially diluted standards were added to antibody-precoated wells and incubated for 2 h at room temperature. After washing it, biotinylated detection antibodies and streptavidin-HRP conjugates were sequentially added. TMB substrate was incubated for 20 min, and the absorbance was measured using microplate reader.

**qRT-PCR Analysis of Cells**

Total RNA was extracted from RAW264.7 and BMDMs using TRIzol® reagent (Invitrogen). Briefly, cells were treated with TRIzol, homogenized with chloroform, and precipitated with isopropanol. RNA pellets were washed with 75% ethanol, air-dried, and dissolved in RNase-free water. RNA concentration and purity were determined using NanoDrop 2000 (Thermo Scientific). Then the cDNA was synthesized using PrimeScript™ RT Reagent Kit (Takara). After that, primers for M1 markers (TNF-α, CD86, iNOS), M2 markers (Fizz1, Arg1, CD206), mitochondrial integrity markers (SIRT1, PGC-1α, TFAM), and GAPDH were designed and synthesized by Takara Bio. qRT-PCR was performed using TB Green® Premix Ex Taq™ II (Takara) on Bio-Rad Real-Time System.

Primer sequences we used in this study were as follows: *GAPDH*, 5’TGAGGTGACCGCATCTTCTTG3’ and 5’TGGTAACCAGGCGTCCGATA3’; *CD86*, 5’CTTACGGAAGCACCCACGAT3’ and 5’CGGCAGATATGCAGTCCCAT3’; *TNF-α,* 5’GATCGGTCCCCAAAGGGATG3’ and 5’CCACTTGGTGGTTTGTGAGTG3’; *iNOS,* 5’TCACCTTCGAGGGCAGCCGA3’ and 5’TCACCTTCGAGGGCAGCCGA3’; *Arg*, 5’GGAATCTGCATGGGCAACCTGTGT3’ and 5’AGGGTCTACGTCTCGCAAGCCA3’; *Fizz1*, 5’GGGATGACTGCTACTGGGTG3’ and 5’TCAACGAGTAAGCACAGGCA3’; *CD206*, 5’AGACGAAATCCCTGCTACTG3’ and 5’CACCCATTCGAAGGCATTC3’; *Caspase3*, 5’GAGCTTGGAACGGTACGCTA3’ and 5’GAGTCCACTGACTTGCTCCC3’; *Caspase8*, 5’CAGGAGACCATCGAGGATGC3’ and 5’CCCACCGACTGATGTGGAAA3’; *SIRT1*, 5’ CGGCTACCGAGGTCCATATAC3’ and 5’ACAATCTGCCACAGCGTCAT3’; *TFAM,* 5’GAGCGTGCTAAAAGCACTGG3’ and 5’CCACAGGGCTGCAATTTTCC3’; *PCG-1α,* 5’GTTGCCTGCATGAGTGTGTG3’ and 5’TAGAGACGGCTCTTCTGCCT3’; *DRP1,* 5’TGCAGGACGTCTTCAACACA3’ and 5’GACCACACCAGTTCCTCTGG3’; *FDX1,* 5’GCAAGCTCATTGTGTCAGGC3’ and 5’TAGGGTGTGGCTCGGTGATA3’; *LIAS,* 5’GGGTCCCGGACAAGAGTACA3’ and 5’ACGGAGAGCTTTCAGTGTGG3’.

**Immunofluorescence Staining of Macrophage Polarization Markers**

BMDMs cultured on PTCu@GOx-coated porous titanium disks were fixed with 4% paraformaldehyde, permeabilized with 0.1% Triton X-100 for 10 min. Then cells were incubated overnight at 4°C with primary antibodies against CD86 (M1 marker, abcam) and CD206 (M2 marker, abcam) diluted in PBS containing 1% BSA. After washing, samples were incubated with Alexa Fluor 488 and Alexa Fluor 594 (Zhongshanjinqiao, China) for 1 h. Then sealed with Antifade Mounting Medium with DAPI (Beyotime, China). The images were acquired using Leica confocal microscope. ImagJ software was used for semi-quantitative analysis.

**Dot Blot Analysis**

Protein expression levels of inflammatory mediators in macrophages were assessed using the ARY006 Inflammation Panel Dot Blot Kit (R&D, USA) following the manufacturer’s protocol. Briefly, cell lysates were loaded onto nitrocellulose membranes pre-spotted with multiple antibodies targeting different types of inflammatory markers. Membranes were blocked for 1 h and incubated with antibodies cocktail (1:1,000) overnight. After washing, streptavidin-HRP was added and incubated with membranes for 1 h, and then the signals were visualized using chemiluminescence imaging system (BIO-RAD, USA). ImageJ was used for semi-quantitative analysis.

**Western Blot**

In order to evaluate the protein expression level of AMPK and pAMPK in macrophages, western blot was carried out. Briefly, the protein lysates from macrophages were prepared using RIPA. Protein concentrations were determined via BCA assay (Pierce™), and 30 μg of protein per sample was separated on 12.5% SDS-PAGE gels and transferred to PVDF membranes. Next, membranes were blocked with 5% non-fat milk in TBST (Tris-buffered saline with 0.1% Tween 20) for 1 h and incubated overnight at 4°C with following primary antibodies: AMPKα, pAMPKα (1:1000, Proteintech, China), and β-actin (1:5000, Proteintech, China). After washing, membranes were incubated with HRP. Signals were visualized and imaged on a BIO-RAD Imaging System.

**Cytoskeletal Staining**

Macrophages were co-cultured with *S. aureus* on PTCu@GOx-coated porous Ti disks for 12 h. They were irradiated with NIR light (808 nm, 1.5 W/cm²) for 15 min and further incubated for 12 h. After that, the macrophages were fixed with 4% paraformaldehyde and blocked with 5% BSA for 1 h. Then, F-actin filaments were stained with Phalloidin-SF488 (Solarbio, China), and the nuclei were counterstained with DAPI. Confocal microscope was used for imaging. ImageJ software was used for semi-quantitative analysis.

**TEM analysis for Host Cells Mitochondrial Dynamics**

Macrophages were fixed in 2.5% glutaraldehyde, dehydrated via ethanol gradients, and embedded in Epon 812 resin. Ultrathin sections (70 nm) were stained with uranyl acetate and lead citrate. Mitochondrial ultrastructure (fusion/fission) was imaged using a Hitachi TEM at 100 kV.

**JC-1 Staining**

Macrophages cultured on PTCu@GOx-coated titanium were stained with JC-1 dyes (Thermo Fisher Scientific, USA) following the introduction of manufacturer. Briefly, cells were firstly incubated with JC-1 agents (5 μg/mL) for 30 min. Next, samples were washed twice with PBS and counterstained with DAPI (1 μg/mL, 10 min). Confocal microscopy was used for imaging and ImageJ software was used for co-localization analysis.

**MitoTracker and 8-OHdG Co-Staining**

Macrophages cultured on PTCu@GOx-coated porous Ti disks were firstly incubated with MitoTracker Red CMXRos (Beyotime, China), followed by fixation with 4% paraformaldehyde and permeabilization with 0.1% Triton X-100 for 10 min. Oxidized mitochondrial DNA (mtDNA) was next stained using anti-8-OHdG antibody (abcam, USA). Besides, the nuclei were counterstained with DAPI (MCE, USA). Confocal microscope was used for imaging. ImageJ software was used for semi-quantitative analysis.

***In vivo* analysis**

All the *in vivo* experiments had complied with the ARRIVE guidelines and had been approved (CQHS-REC-2020(LSNo.75)) by the Ethics Committee of School of Stomatology, Chongqing Medical University. For diabetic model building, the streptozotocin (STZ) was injected (30 mg/kg) for SD rats and New Zealand Rabbits. The combined injection of STZ and alloxan (30 mg/kg, 50mg/kg, successively) was applied on Beagle dogs.

**SD Rat Subcutaneous Implantation**

Diabetic SD rat model was firstly created, then two 1 cm vertical incisions were made along the midline after skin preparation. simulate infection, 20 μL of MRSA suspension (1 × 10⁶ CFU/mL) was injected into the peri-implant space. Implants in the DM+*MRSA*+PTCu(+) and DM+*MRSA*+PTCu@GOx(+) groups were irradiated with 808 nm NIR light for 15 min. At 14 days post-surgery, rats were sacrificed via CO₂ asphyxiation and the peri-implant soft tissues were collected.

**New Zealand Rabbit Femur Metaphyseal Infectious Bone Defect Model**

After 7 days of diabetic model creation, a 3 mm diameter cylindrical bone defect was surgically created in the metaphyseal region of the femur of rabbits. Then 3D-printed porous titanium implant (3 mm diameter × 8 mm length) with coating was fit into the defect. For infection groups, 20 μL of MRSA suspension (1 × 10⁶ CFU/mL) was injected into the defect site. In the DM+MRSA+PTCu@GOx(+) group, implants were irradiated with NIR light for 15 min post-surgery. 8 weeks after surgery, animals were sacrificed and the femur bone samples were collected for microCT and hard tissue sectioning.

**Beagle Dog Tooth Extraction and Implantology**

For tooth extraction, PM2, PM3, PM4 and M2 of each side of beagle dogs were bilaterally hemisected with dental burs and distal roots were extracted atraumatically. The extraction wounds were closed using interrupted sutures. The mesial roots were retained and conducted with root canal therapy. After 12-week healing, STZ and alloxan were injected for diabetic model building. At 4 weeks post-injection, dental implants (3.5 mm diameter, 10 mm length, Rui Qi, China) were implanted into each extraction site according to the introduction of manufacturer and gingival closure was performed. After 8 weeks, *P. gingivalis* was applied at peri-implant area for diabetic implantitis (DPI) making. After another 8 weeks, animals were sacrificed and the alveolar bone samples were collected with implants.

**Blood Biochemical Analysis**

Blood samples were collected through cardiac puncture and the serum was separated by centrifugation (3,000g, 15 min, 4°C). The biochemical parameters were then tested using Automatic Biochemical Analyzer (Mindray, China), such as white blood cell count (WBC), neutrophil percentage (NE), lymphocyte percentage (LY), aspartate aminotransferase (AST), alkaline phosphatase (ALP) and so on.

***In vivo* Stability Assessment**

Using the ​​New Zealand rabbit femoral metaphyseal model​​, PTCu@GOx(+)-coated scaffolds were explanted 14 days post-implantation. Post-sonication (30 min), ​​ICP-OES​​ was used to reveal the residual Cu​​ within the scaffold, further supporting the coating stability *in vivo*.

**Histology and Immunofluorescence Staining**

The H&E staining Kit, Giemsa staining Kit and Masson trichrome staining Kit (Solarbio, China) were used following the introduction of manufacturer. As for immunofluorescence, sections were deparaffinized in xylene and rehydrated through graded ethanol. After antigen retrieval, they were blocked with 5% goat serum in PBS for 1 h and incubated with primary antibodies of CD31 and α-SMA overnight at 4°C. Next day, after washing with PBS, sections were incubated with Alexa Fluor 488 (Zhongshanjinqiao, China) and Alexa Fluor 594 (Zhongshanjinqiao, China) for 1 h at room temperature. Moreover, the nuclei were counterstained with DAPI and confocal microscopy was used for imaging.

**Agar Assay and CFU Counting *in vivo***

Peri-implant liquid was harvested from the subcutaneous site of SD rats and the peri-implant area of Beagle dogs. The samples were then diluted in PBS to 10⁻⁶ and was spread on agar plates. Next, the agar plates were incubated at 37°C for 24 h, and CFUs were manually counted.

**Van Gieson (VG) Staining**

First with preheated methylene blue solution (60°C, 15 min) to highlight mineralized bone and titanium interfaces, followed by rinsing in warm distilled water. Sections were then stained with acid fuchsin-picric acid solution (5 min) to visualize collagen fibers. All sections were then air-dried for 24 h and resin-mounted. VS200 digital slide scanner (OLYMPUS, USA) was used to capture images of all the stained sections.

**Statistical Analysis**

All the data collected in this study were analyzed using SPSS 23.0 (IBM, USA). Moreover, one-way ANOVA with Student-Newman-Keuls post hoc test was applied for multi-group comparisons. Significance was determined as *: *p* < 0.05 and ****: *p* < 0.01.

**Results**


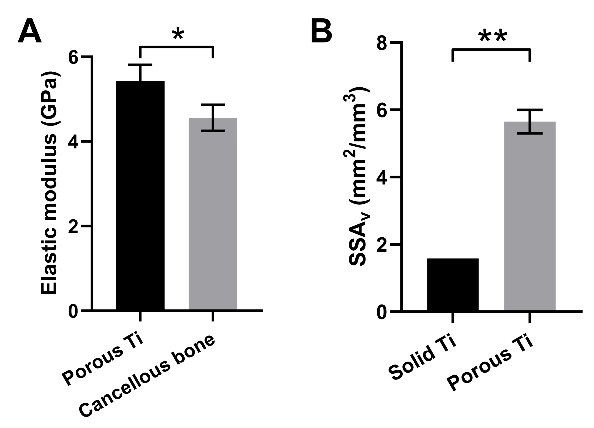


**Figure S1. Physical properties of 3D printed porous Ti implants.** (A) Compressive strengthen comparison of 3D printed porous Ti and cancellous bone; (B) Calculation of the specific surface area of solid Ti implant and 3D printed porous Ti implants based on high-resolution microCT result. (**p*<0.05; ***p*<0.01)


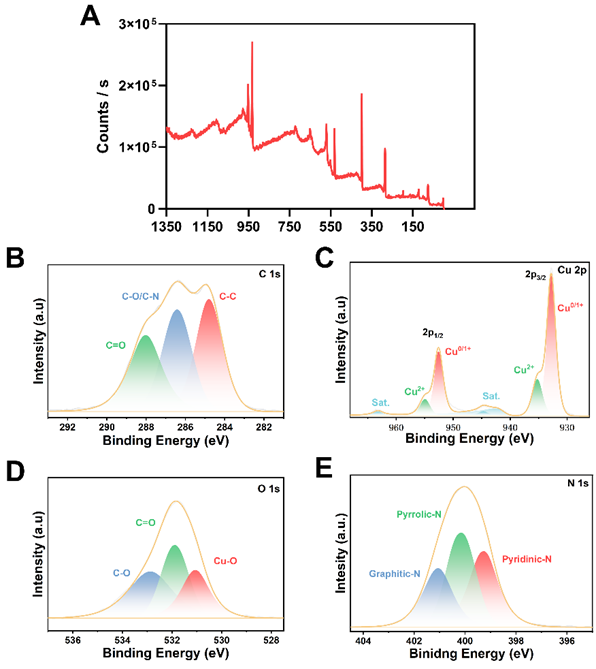


**Figure S2. High-resolution XPS result of Cu-CQDs.** (A) XPS survey scan; (B-E) High-resolution deconvoluted XPS spectra of (B) C 1s, (C) Cu 2p, (D) O 1s, and (E) N 1s regions.


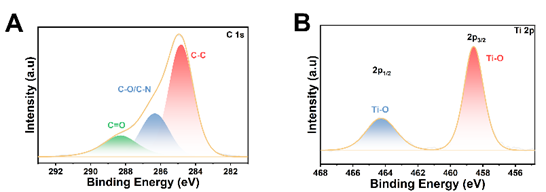


**Figure S3.** High-resolution XPS spectra of PTCu@GOx coating on porous Ti disks of (A) C 1s and (B) Ti 2p regions.


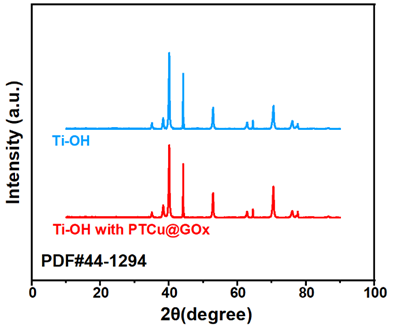


**Figure S4.** XRD analysis of the alkali-heat-treated porous Ti disks and the PTCu@GOx-coated alkali-heat-treated porous Ti disks.


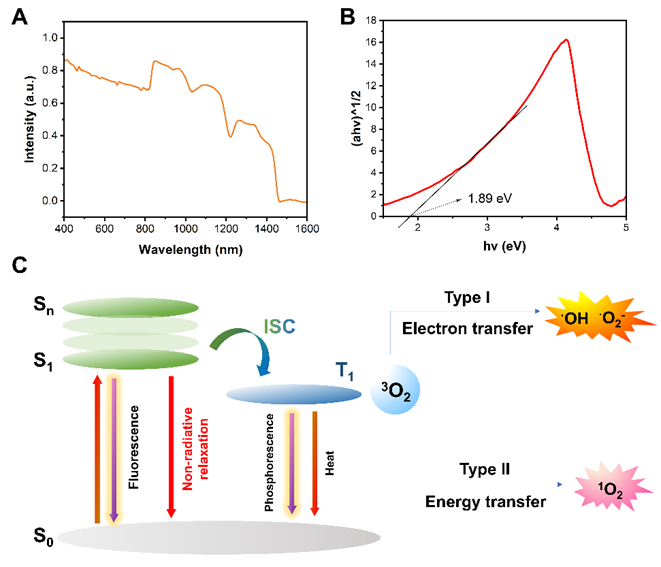


**Figure S5.** (A-B) UV-Vis survey spectra and corresponding band gap calculations. (C) Schematic illustration of the mechanism for ROS generation under NIR excitation: Electrons are photoexcited to the singlet state, undergo intersystem crossing to the triplet state, and subsequently produce distinct ROS via Type I (electron transfer) and Type II (energy transfer) pathways.


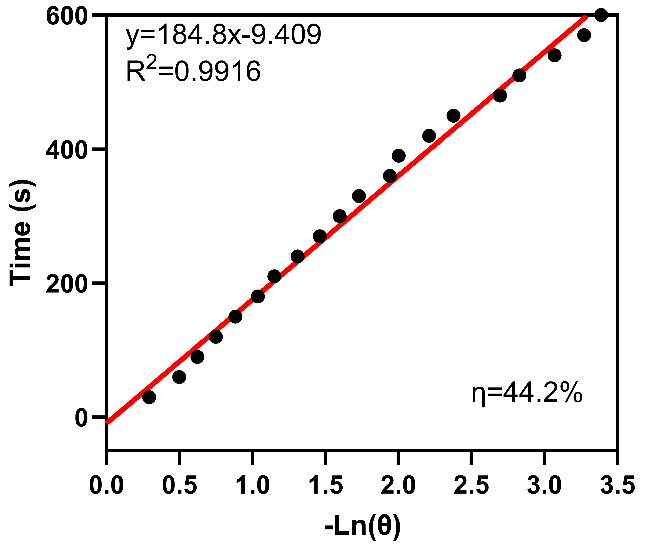


**Figure S6.** Calculation of the photothermal conversion efficiency of the PTCu@GOx coating under NIR irradiation.


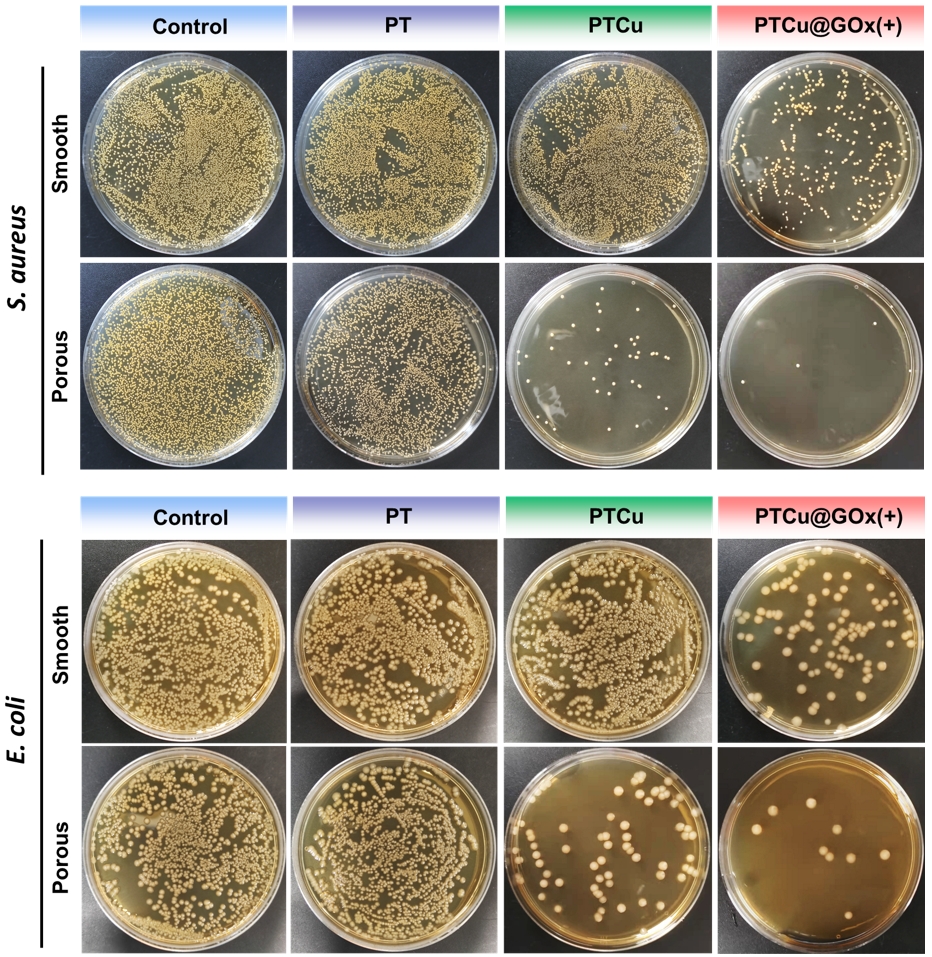


**Figure S7.** Smooth and porous Ti disks were treated with PT, PTCu, and PTCu@GOx(+) (where "+" indicates NIR stimulation). *S. aureus* and *E. coli* were selected as the model bacterial strains. After co-incubation with the materials, bacterial viability was assessed through agar plating and CFU counting.


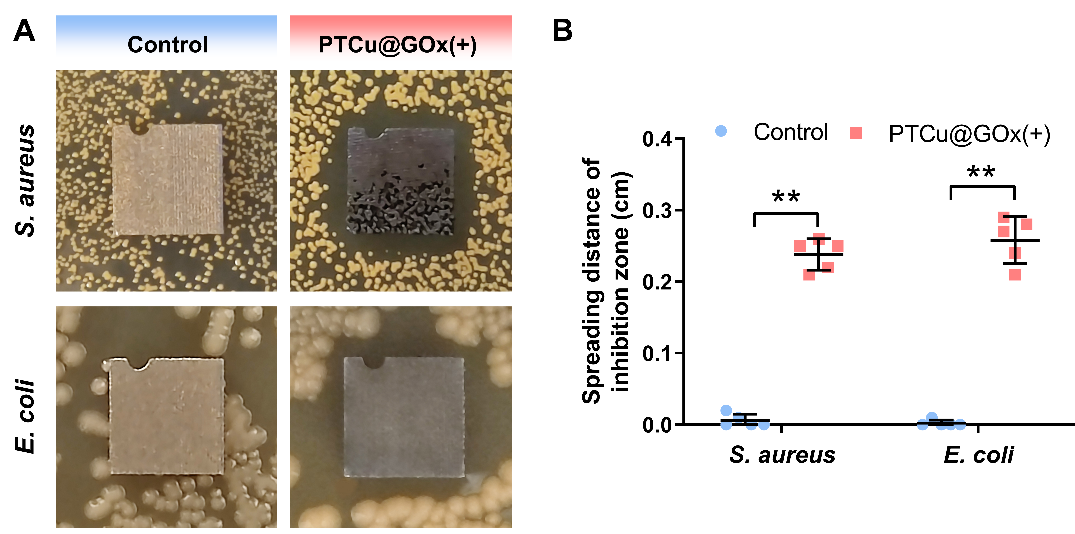


**Figure S8.** (A) Inhibition zone assay of *S. aureus* and *E. coli* to evaluate the antibacterial performance of PTCu@GOx coating on porous titanium sheets; (B) Measurement and quantitative analysis of inhibition zone diameters. (**p*<0.05; ***p*<0.01)


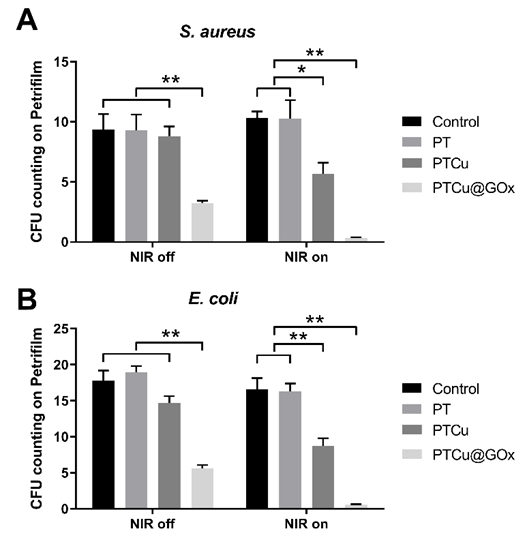


**Figure S9.** Petrifilm detection with PTCu@GOx-coated porous titanium sheets, with the contact antibacterial efficacy against (A) *S. aureus* and (B) *E. coli* being observed respectively. (**p*<0.05; ***p*<0.01)


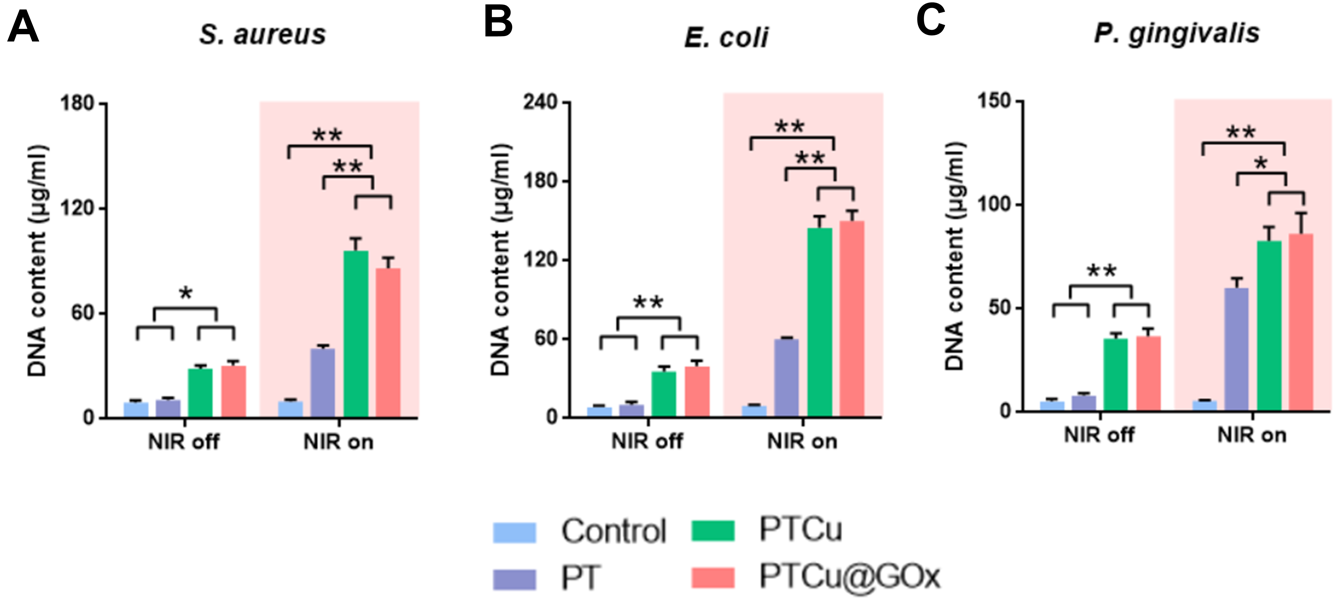


**Figure S10.** Nucleic acid leakage detection results showing the antibacterial function of PTCu@GOx drug reservoir on (A) *S. aureus*, (B) *E. coli*, and (C) *P. gingivalis*. (**p*<0.05; ***p*<0.01)


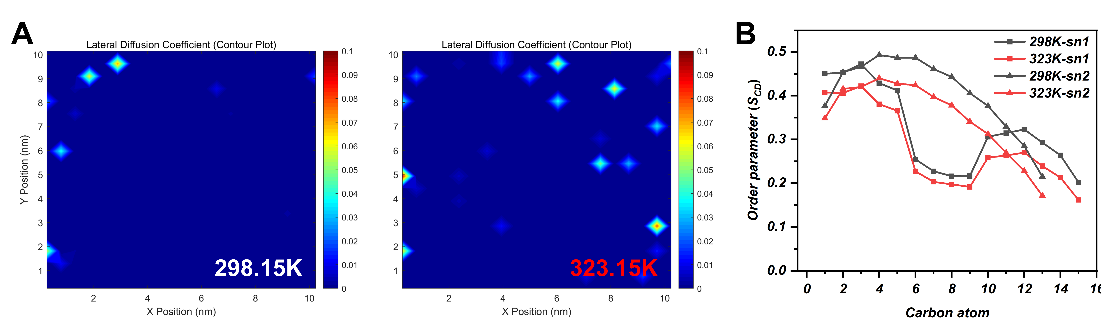


**Figure S11.** (A) Analysis results from molecular dynamics simulations. Lateral diffusion rates of the bilayer at 298 K and 323 K; (B) Lipid tail chain order parameters for POPG membrane lipids at different temperatures.


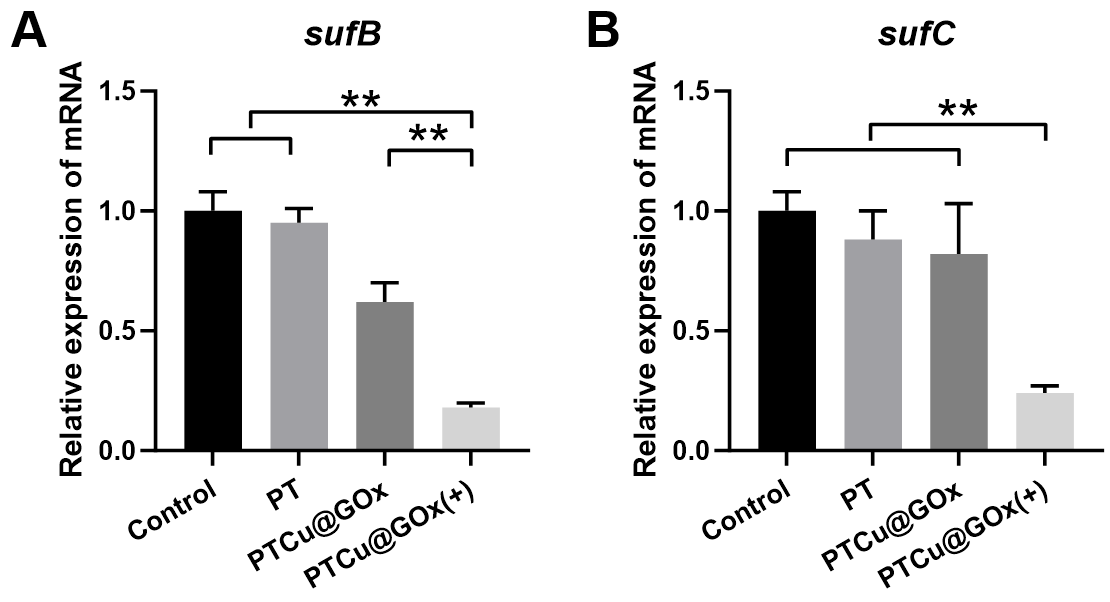


**Figure S12.** qRT-PCR results for MRSA showing the expression levels of iron-sulfur cluster-related biomarker genes *sufB* (A) and *sufC* (B). (**p*<0.05; ***p*<0.01)


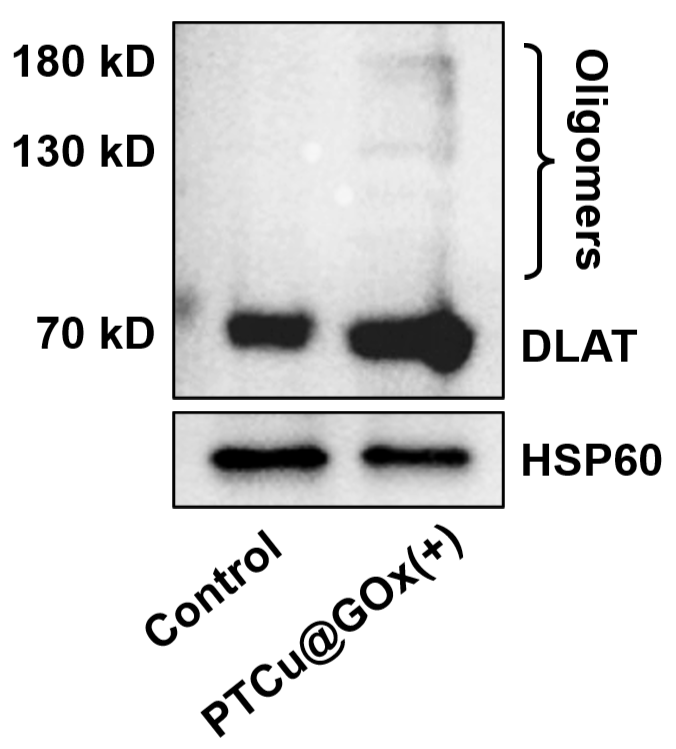


**Figure S13.** Western blot of DLAT oligomerization of MRSA treated with PTCu@GOx. HSP60 was used as the loading control.


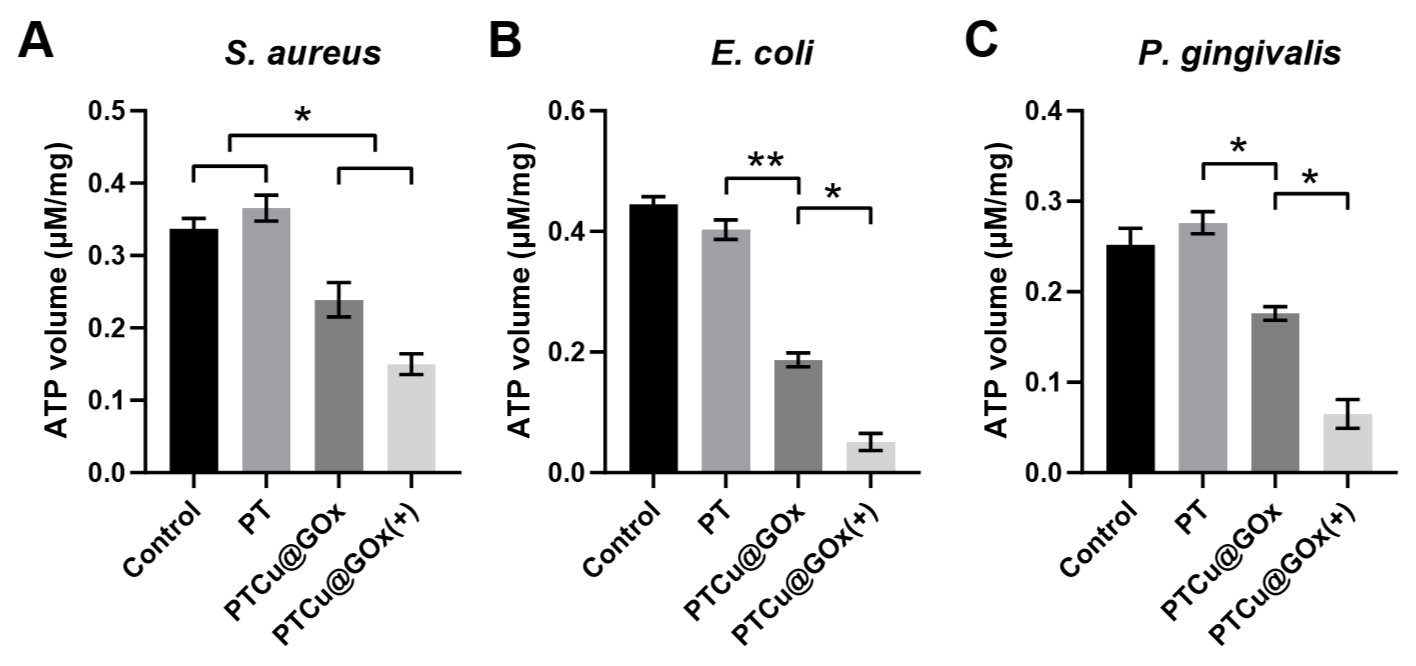


**Figure S14.** ATP assay reflecting the energy generation of S. aureus, E. coli, P. gingivalis treated with PTCu@GOx. (**p*<0.05; ***p*<0.01)


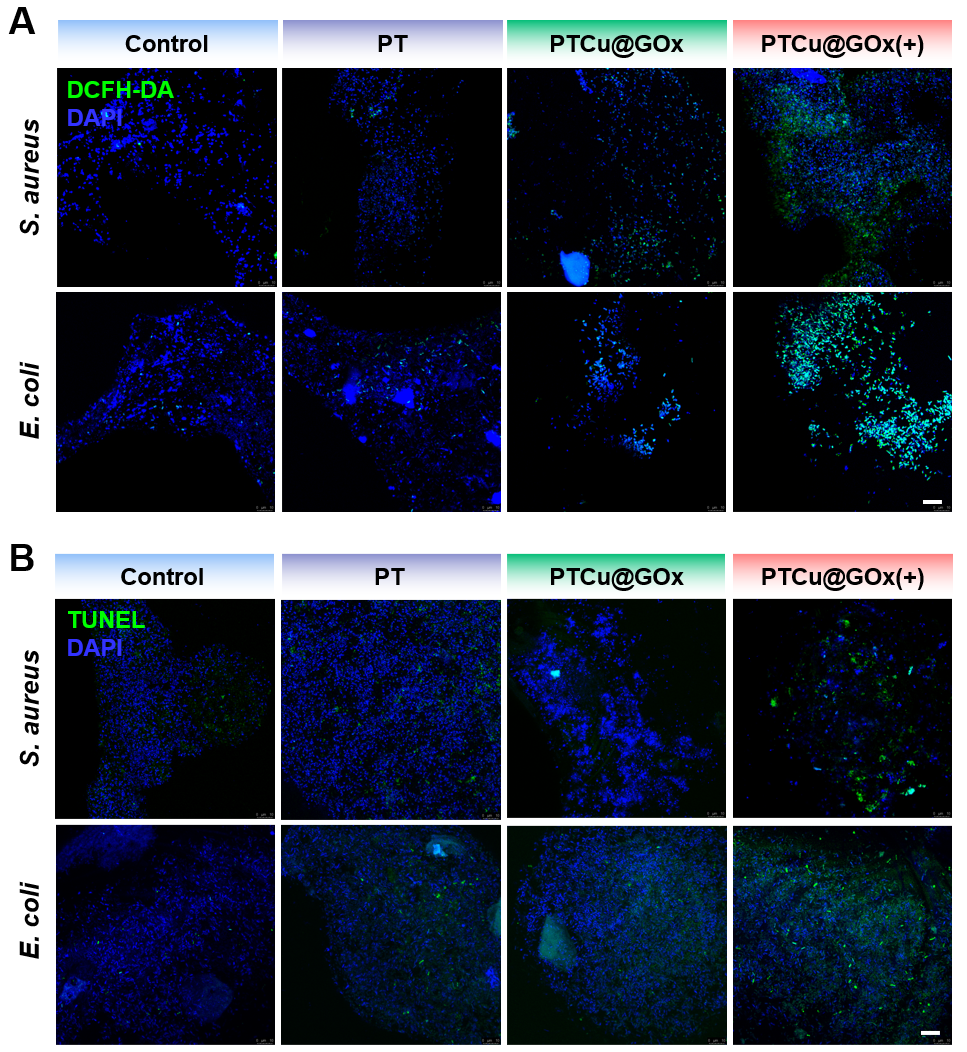


**Figure S15. Fluorescence probe staining results for bacterial ROS production and apoptosis markers.** (A) DCFH-DA staining indicating total ROS generation levels; (B) TUNEL assay showing bacterial apoptosis outcomes under different treatments. (Scale bar = 50 μm)


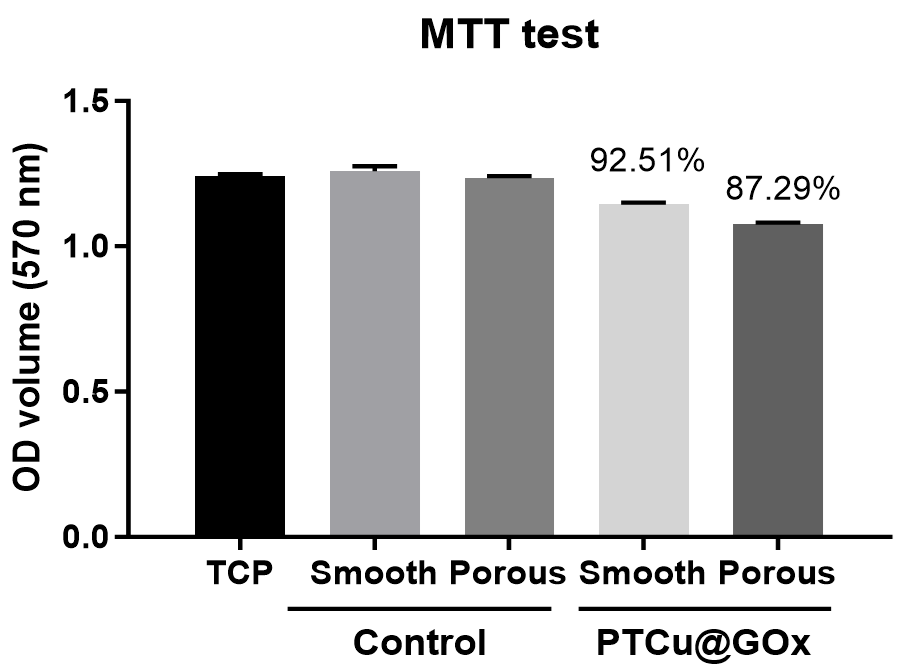


**Figure S16.** MTT test showing the biocompatibility of PTCu@GOx coating on smooth and porous Ti disks by culturing macrophages on the materials.


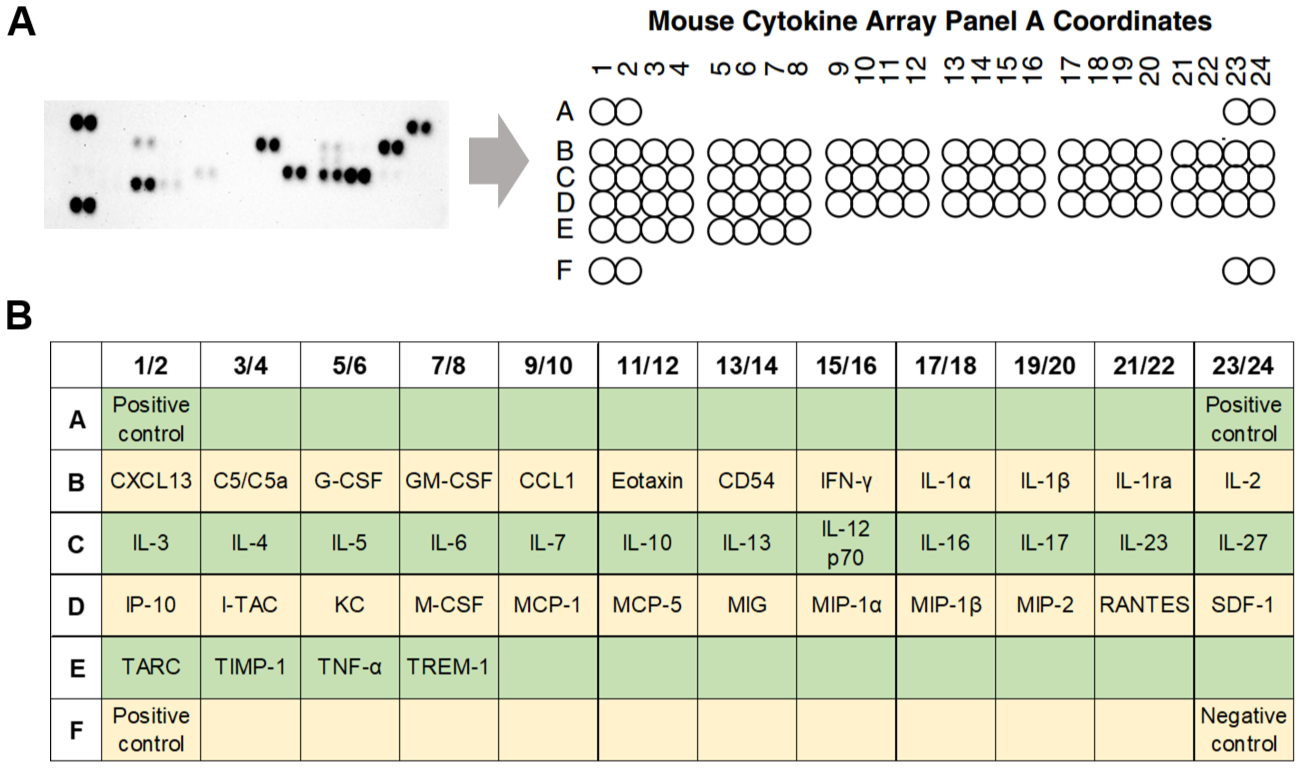


**Figure S17.** Cytokine array panel showing the target markers of inflammation in dot blot test.


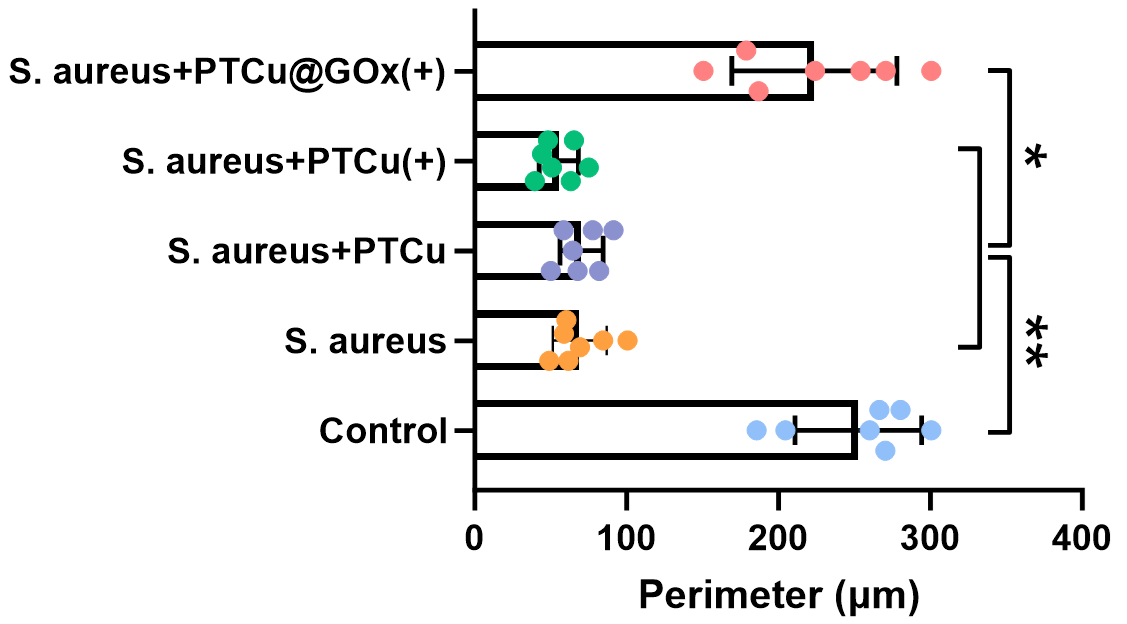


**Figure S18.** Semi-quantitative analysis of the perimeters of BMDMs co-cultured with *S. aureus* on PTCu@GOx coated porous Ti disks, showing the cell spreading capability of cells and indicating the cytoprotection potential of PTCu@GOx in infectious environment. (**p*<0.05; ***p*<0.01)


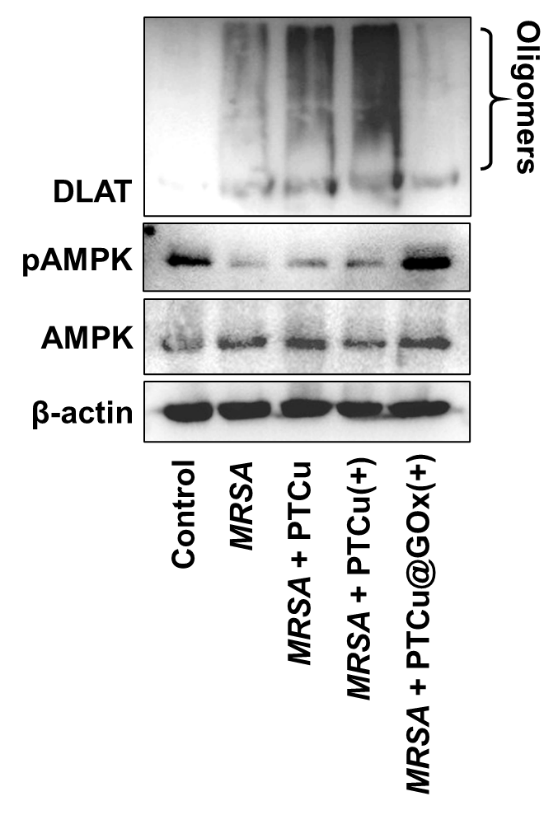


**Figure S19.** Western blot of AMPK​​, ​​pAMPK, and ​​DLAT oligomerization of macrophages co-cultured with MRSA and treated with PTCu@GOx.


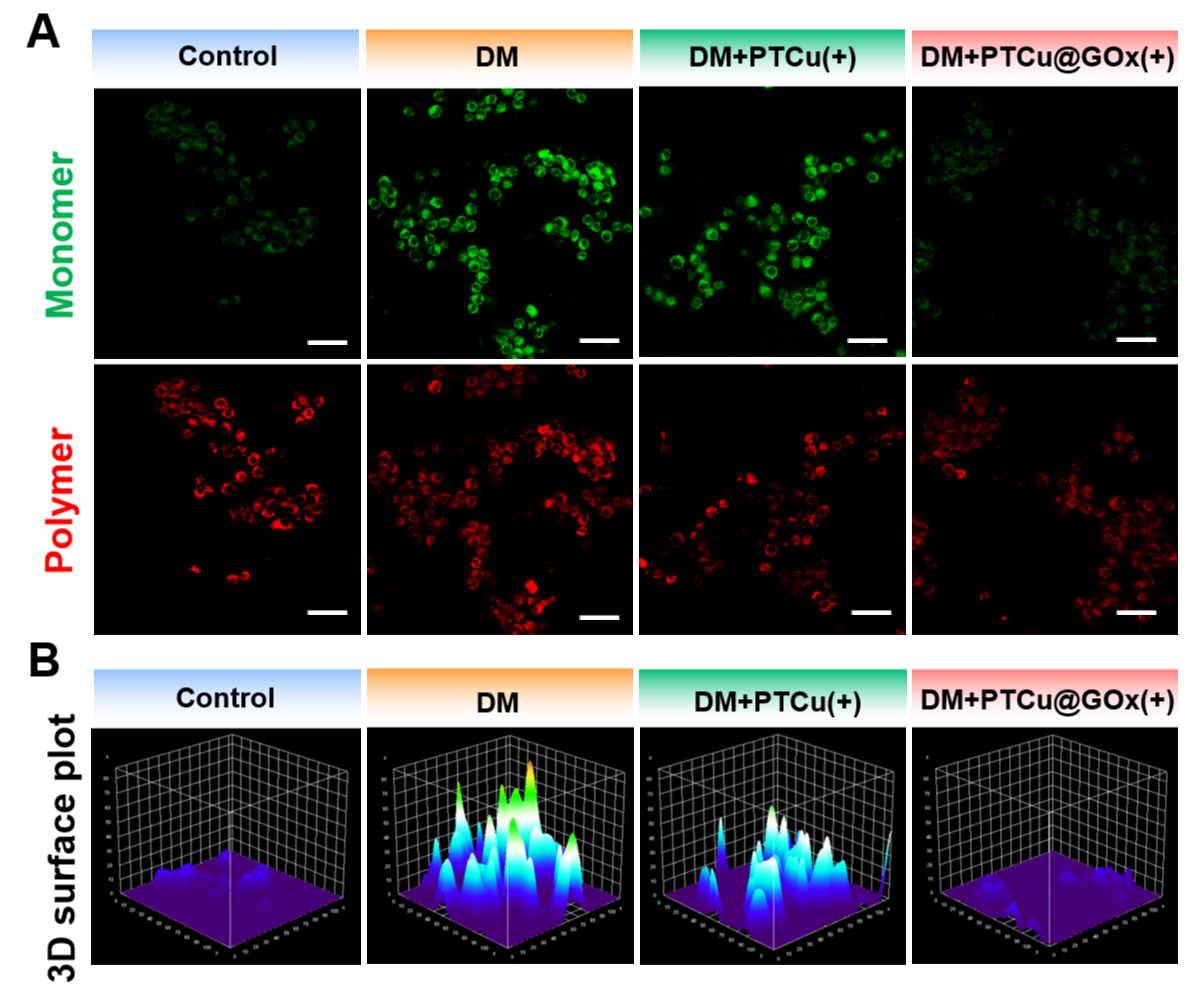


**Figure S20.** (A) JC-1 staining and (B) semi-quantification of 3D surface plot for BMDMs cultured on PTCu@GOx-coated porous Ti disks. (Scale bar = 50 μm)


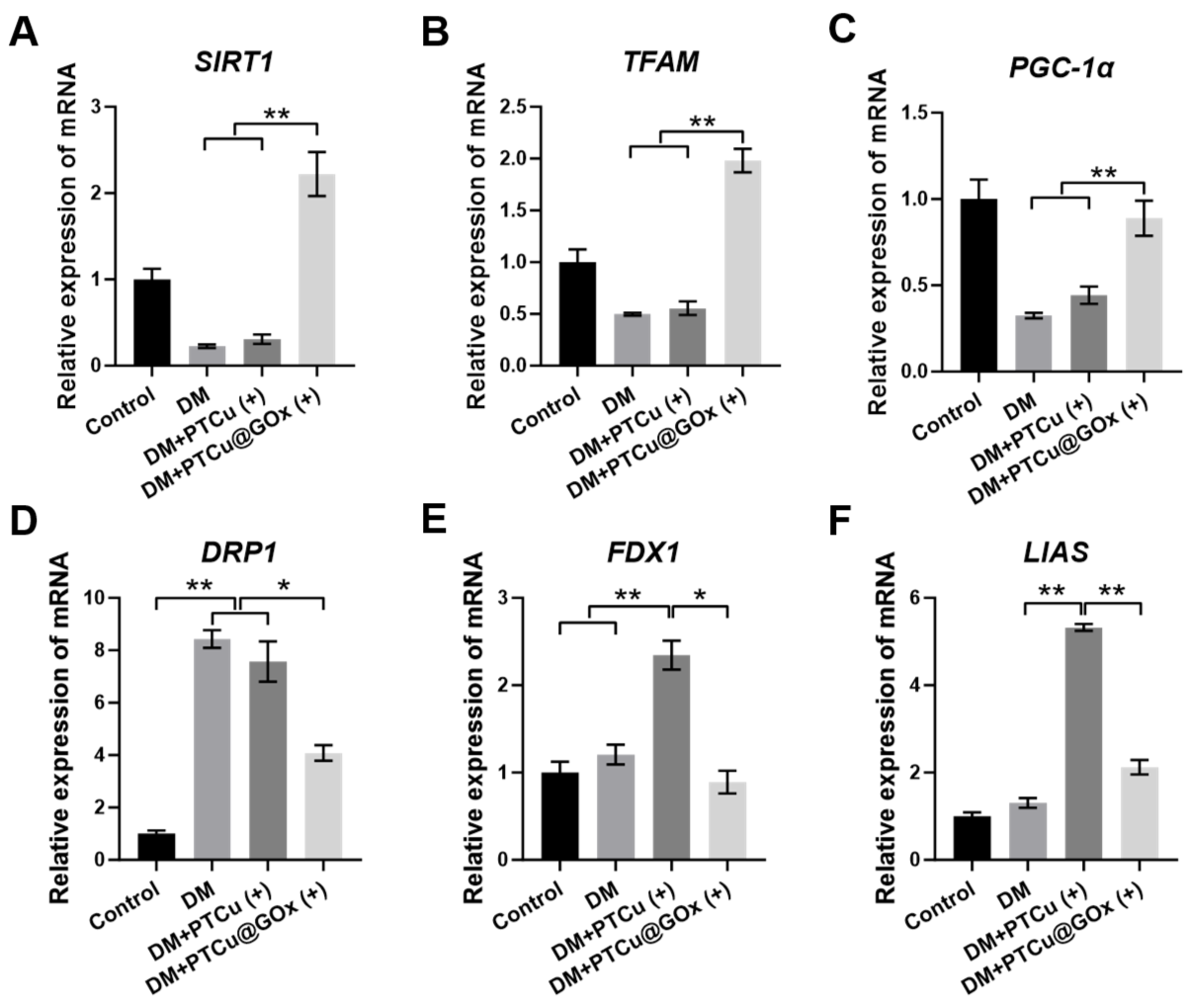


**Figure S21.** qRT-PCR results of BMDMs cultured on coated titanium sheets, including (A-D) genes related to mitochondrial quality control and function (*SIRT1, TFAM, PGC-1α, DRP1*) and (E-F) key genes involved in cuproptosis (*FDX1, LIAS*). (*p<0.05; **p<0.01)


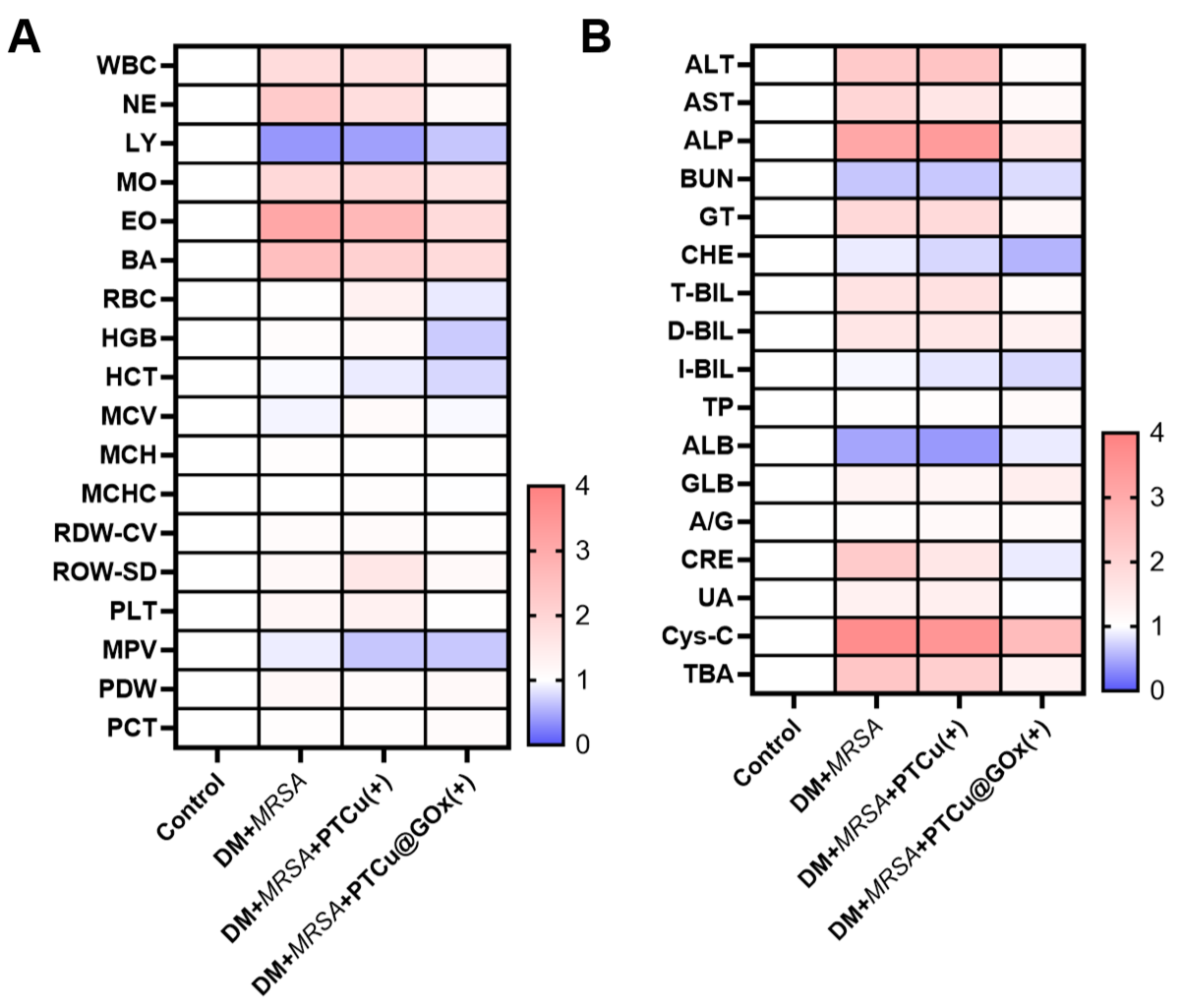


**Figure S22.** (A) Blood biochemical and (B) liver and kidney function indices in healthy and diabetic infectious SD rat model with or without PTCu@GOx treatment.


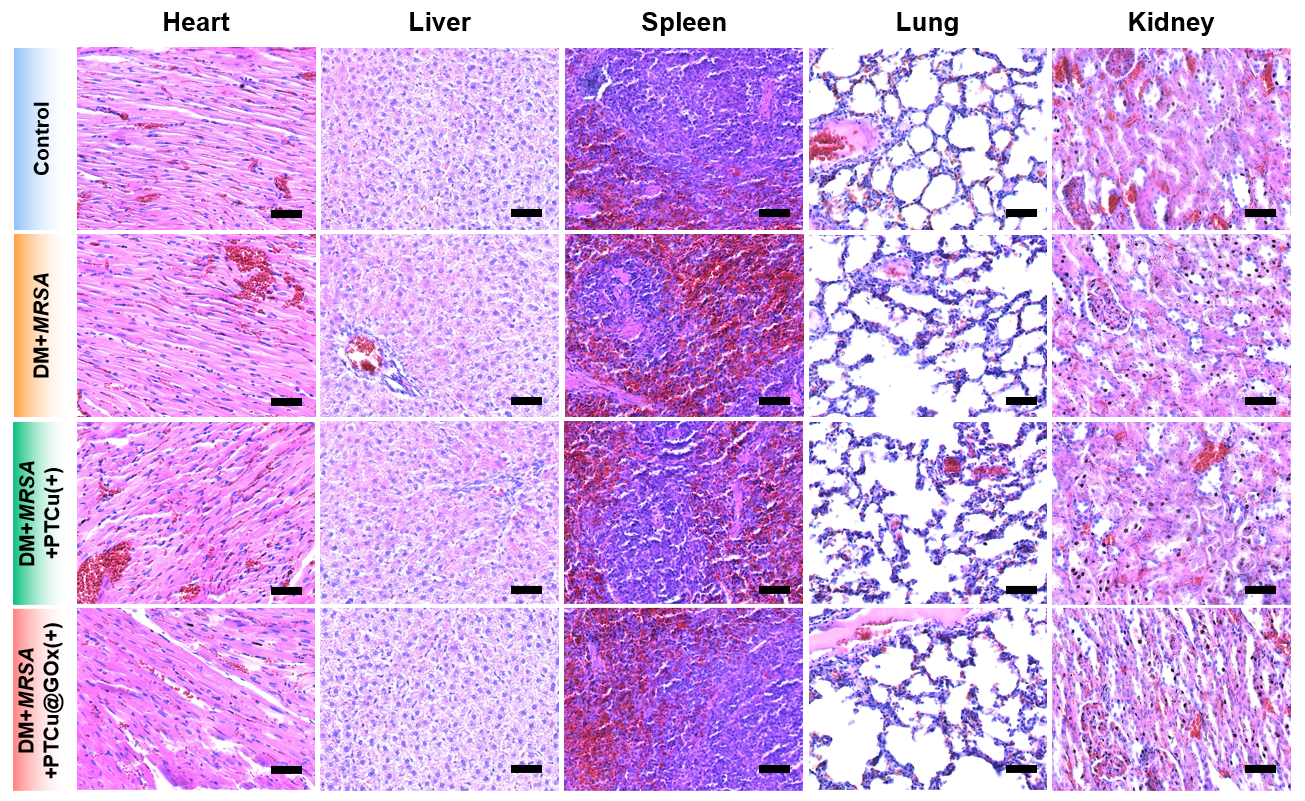


**Figure S23.** The H&E staining of heart, liver, spleen, lung and kidney of SD rats from different groups showing the biosafety of PTCu@GOx drug reservoir. (Scale bar = 50 μm)


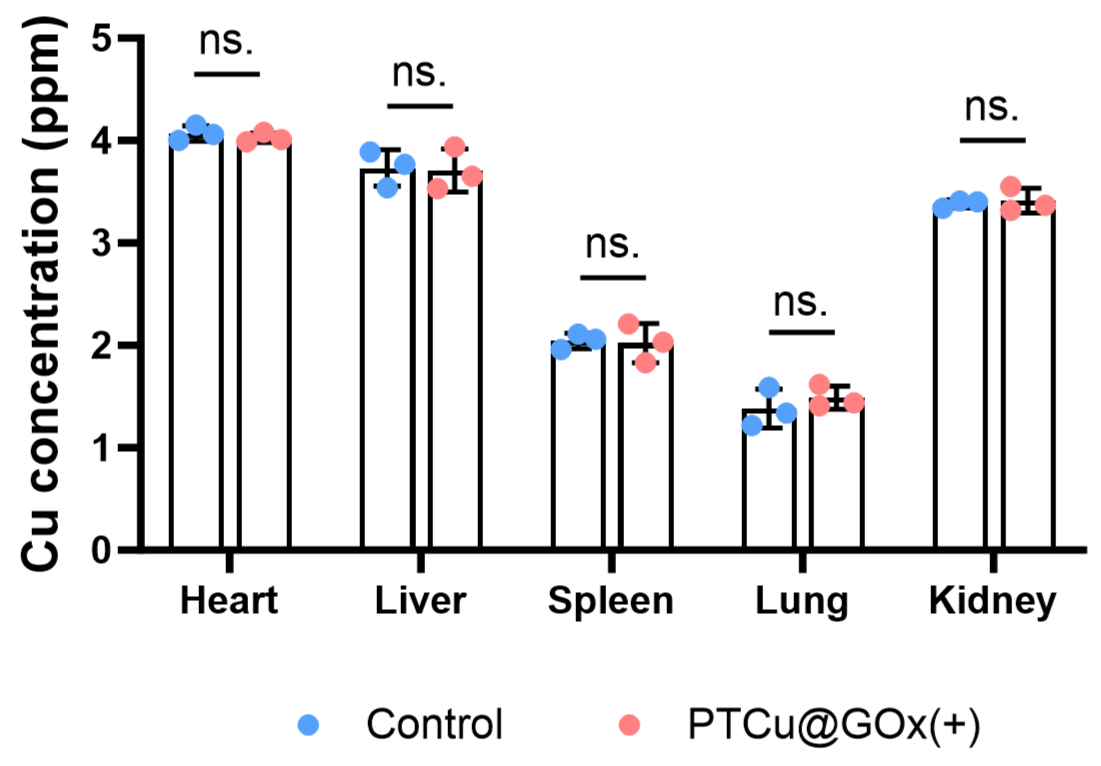


**Figure S24.** ICP-OES results showing the Cu concentration within different organs in New Zealand rabbits with the PTCu@GOx coated porous implants applied at metaphysis of femoral.


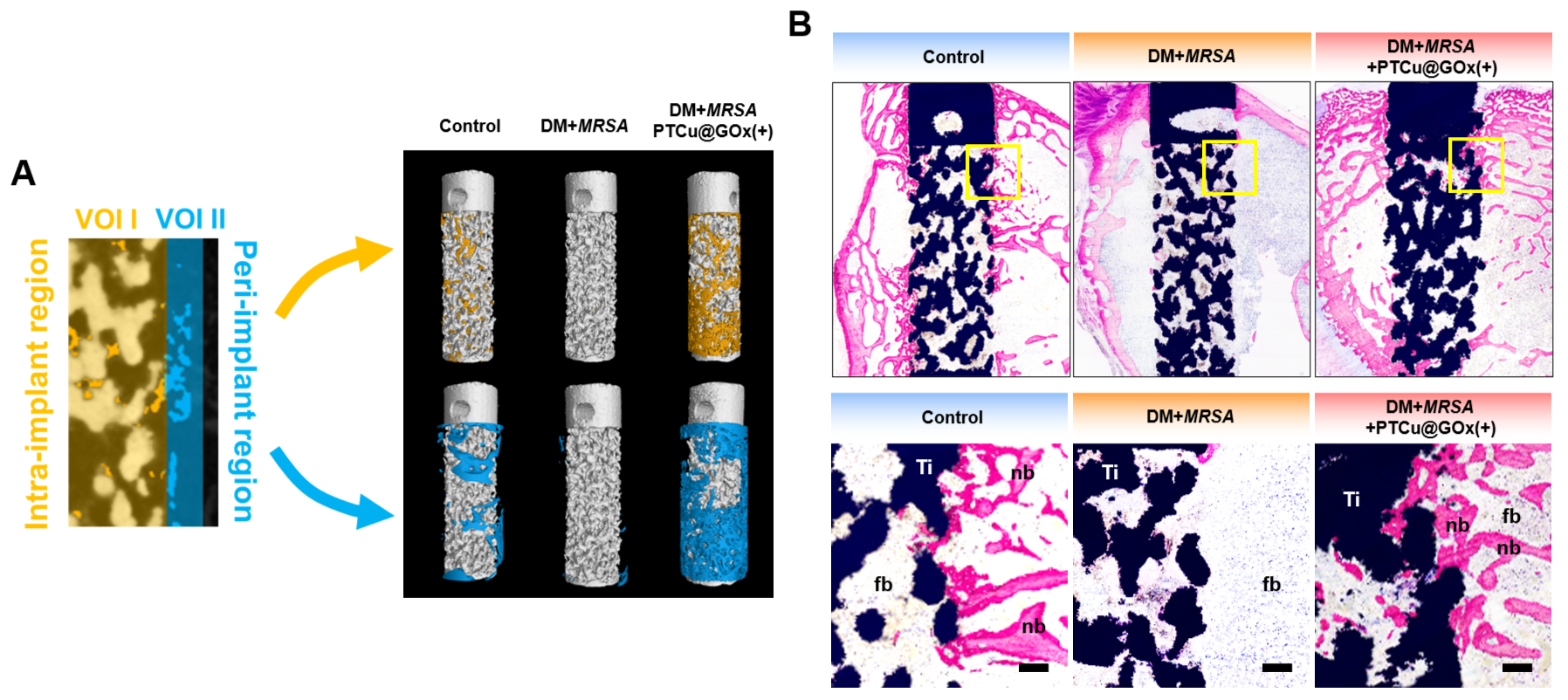


**Figure S25.** The microCT and histological results of infectious bone defect healing in New Zealand Rabbit femur defect model treated with porous implant with PTCu@GOx. (A) the 3D reconstruction result of VOI I and VOI II reagions; (B) the VG staining image of peri-implant region showing the bone defect healing and new bone ingrowth into porous Ti implants. (Scale bar = 100 μm)


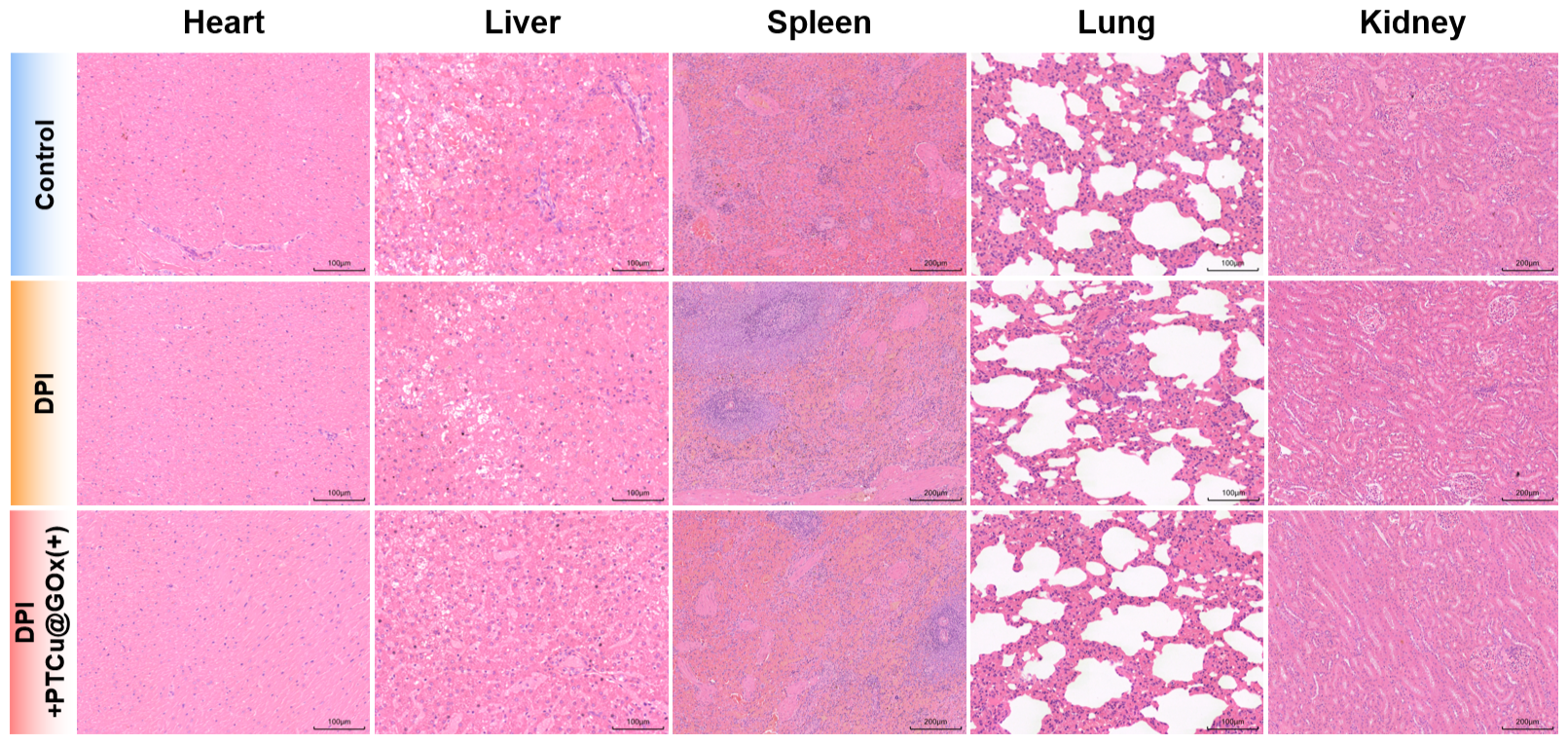


**Figure S26.** The H&E staining of heart, liver, spleen, lung and kidney of beagle dogs from different groups showing the biosafety of PTCu@GOx drug reservoir on commercial implants. (Scale bar = 100 or 200 μm)


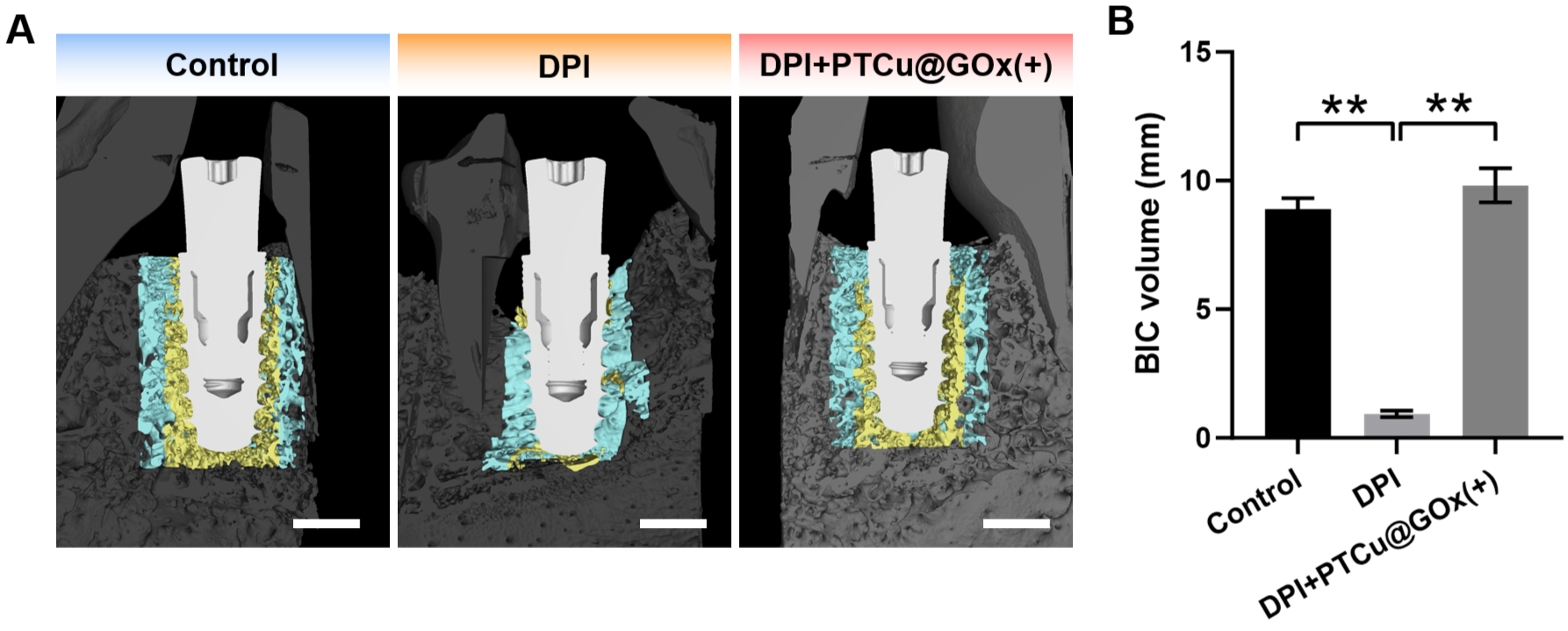


**Figure S27.** The microCT result and semi-quantitative analysis of bone-implant-contact (BIC). (A) microCT reconstruction of commercial implant and surrounding bone tissue (Scale bar = 3 mm); (B) the BIC volume of different groups. (*p<0.05; **p<0.01)
